# Viral entry and translation in brain endothelia provoke influenza-associated encephalopathy

**DOI:** 10.1007/s00401-024-02723-z

**Published:** 2024-04-30

**Authors:** Shihoko Kimura-Ohba, Mieko Kitamura, Yusuke Tsukamoto, Shigetoyo Kogaki, Shinsuke Sakai, Hiroaki Fushimi, Keiko Matsuoka, Makoto Takeuchi, Kyoko Itoh, Keiji Ueda, Tomonori Kimura

**Affiliations:** 1 Division of Virology, Department of Microbiology and Immunology, Osaka University Graduate School of Medicine, 2-2 Yamada-Oka, Suita, Osaka 565-0871 Japan; 2 Reverse Translational Research Project, Health and Nutrition (NIBIOHN), National Institutes of Biomedical Innovation, Osaka, Japan; 3 KAGAMI Project, Health and Nutrition (NIBIOHN), National Institutes of Biomedical Innovation, Osaka, Japan; 4 Department of Pediatrics and Neonatology, Osaka General Medical Center, Osaka, Japan; 5 Department of Nephrology, Osaka University Graduate School of Medicine, Osaka, Japan; 6 Department of Pathology, Osaka General Medical Center, Osaka, Japan; 7 Department of Pathology, Osaka Women’s and Children’s Hospital, Osaka, Japan; 8 Department of Pathology and Applied Neurobiology, Graduate School of Medical Science, Kyoto Prefectural University of Medicine, Kyoto, Japan

**Keywords:** Influenza-associated encephalopathy (IAE), Influenza A virus, Clasmatodendrosis, Brain endothelial cells, Transcription

## Abstract

Influenza-associated encephalopathy (IAE) is extremely acute in onset, with high lethality and morbidity within a few days, while the direct pathogenesis by influenza virus in this acute phase in the brain is largely unknown. Here we show that influenza virus enters into the cerebral endothelium and thereby induces IAE. Three-weeks-old young mice were inoculated with influenza A virus (IAV). Physical and neurological scores were recorded and temporal-spatial analyses of histopathology and viral studies were performed up to 72 h post inoculation. Histopathological examinations were also performed using IAE human autopsy brains. Viral infection, proliferation and pathogenesis were analyzed in cell lines of endothelium and astrocyte. The effects of anti-influenza viral drugs were tested in the cell lines and animal models. Upon intravenous inoculation of IAV in mice, the mice developed encephalopathy with brain edema and pathological lesions represented by micro bleeding and injured astrocytic process (clasmatodendrosis) within 72 h. Histologically, massive deposits of viral nucleoprotein were observed as early as 24 h post infection in the brain endothelial cells of mouse models and the IAE patients. IAV inoculated endothelial cell lines showed deposition of viral proteins and provoked cell death, while IAV scarcely amplified. Inhibition of viral transcription and translation suppressed the endothelial cell death and the lethality of mouse models. These data suggest that the onset of encephalopathy should be induced by cerebral endothelial infection with IAV. Thus, IAV entry into the endothelium, and transcription and/or translation of viral RNA, but not viral proliferation, should be the key pathogenesis of IAE.

**Supplementary Information:**

The online version contains supplementary material available at 10.1007/s00401-024-02723-z.

## Introduction

Influenza virus infection is often associated with neurological symptoms; i.e., febrile seizure, meningitis, transverse myelitis, Guillain-Barré syndrome, and encephalopathy/encephalitis [20, 22, 100]. Of these, influenza-associated encephalopathy (IAE) is one of the most serious neurological complications, often resulting in severe sequelae with high mortality [11, 63]. Acute encephalopathies, including IAE, had started to be reported in the 1990s mainly in East Asia with pediatric cases [58, 92]. Recently, the number of reports has increased, especially during and after the 2009 pandemic, from all over the world, not only of children, but also of adults to the elderly [6, 8, 22, 31, 57, 64, 72, 73, 83].

Various pathogens, mainly viruses, cause this severe neurological complication [1]. Such cases have been reported as Reye syndrome, influenza encephalitis, and recent neurological complications of coronavirus disease 2019 (COVID-19), which included the broader disease entity of acute encephalopathy and encephalitis related to infection [14, 16, 18, 30, 78, 91, 94, 96]. The most recent national surveillance in Japan pointed out the changes in responsible pathogens; a decrease in influenza virus along with a wider range of pathogens [78].

With the increasing availability of magnetic resonance imaging (MRI), acute encephalopathy has been subclassified into several clinico-radiological syndromes such as acute necrotizing encephalopathy (ANE) [58], hemorrhagic shock and encephalopathy syndrome (HSES) [31, 50], acute encephalopathy with biphasic seizures and late reduced diffusion (AESD) [89], and clinically mild encephalitis/encephalopathy with a reversible splenial lesion (MERS) [87]. Although overall mortality nowadays improved compared to that of twentieth century, there was still no fundamental treatment but supportive care and only 30–50% recovered without sequelae, including mild cases [1, 11, 22, 42].

The pathology of IAE has been investigated in clinical studies and animal experiments, focusing on host factors and biomarkers. Risk genetic polymorphisms have been also reported in East Asian populations. These include *CPT-2*, *RANBP2, SCN1A*, and *TLR-3* genes [7, 28, 33, 38, 47, 71, 79, 81]. Markers of disease severity and treatment efficacy have been examined using physiological, radiological, and biochemical analysis [3, 27, 32, 34, 35, 39, 43, 51, 61]. Several mechanistic studies were performed using IAE related animal models; Reye syndrome-related animal models, congenital metabolic disease-related models, administration of lipopolysaccharides (LPS) or cytokines, models with neurotropic influenza virus, and others [5, 10, 12, 15, 36, 52, 66, 90, 93, 95, 98, 101]. Although these models showed brain edema and increased cytokines, the dynamics and pathogenesis of the influenza virus in IAE are still unknown.

Autopsy research has identified IAE-related pathological features [40, 54, 58, 63, 86, 92, 102]. Brain edema is severe and is sometimes accompanied by herniation. The inflammatory response is inactive, while multiple vascular lesions, such as hemorrhage, bleedings, fibrin thrombosis, and leakage of plasma components, are observed in the brain parenchyma [44, 49, 67]. It is noteworthy that the virus antigen was detected in some cases in the brain parenchyma and spinal fluid [19, 23, 88, 99]. The detection of viral antigen or RNA from edematous brains has been also reported with the influenza virus-inoculated mouse models [80, 101]. Autopsy studies suggested that the virus itself should have a pathological role in IAE, although the virus has hardly been detected in human IAE patients probably due to its rapid and progressive course.

Patients with severe types of IAE develop critical neurological symptoms such as seizures and coma within 10 h after the onset of fever and die within 48 h due to the fatal brain edema [63, 69]. Brain autopsies revealed fewer inflammatory cells and less detection of the virus, as noted above, in contrast to the severe edema and high fever. In the clinical course of IAE, there is a time window between the viral infection and the brain edema, that is, between the infection and the onset of neurological symptoms. This time window is an important indicator of IAE pathogenesis since it must include the key viral dynamics to trigger the brain edema.

To investigate viral dynamics in this time window, we developed an IAE mouse model and analyzed its pathophysiology over time. Furthermore, we compared and verified the results with the involved cell lines and IAE autopsy brains, to confirm the pathogenesis of the infection-related acute brain edema. Through this analysis, we identified the interventional methods to prevent the onset of IAE.

## Materials and methods

Further information can be found in Supplemental Methods.

### Virus

Influenza A/Puerto Rico/8/34 (PR8, H1N1) was obtained from ATCC (VR95; Manassas, VA, USA). To propagate IAV, Madin-Darby canine kidney (MDCK) cells (JCRB9029, NIBIOHN) was inoculated with the virus at an MOI of 0.001 and then cultured in virus culture medium (VCM; minimum essential medium (MEM) containing 0.01 M HEPES, 0.21% albumin, and 0.25% trypsin) in a cell culture incubator at 37 °C and 5% CO_2_ for 72 h. The crude viral lysates of MDCK cells were spun down at 4 °C for 15 min at 4000×*g*. The viral supernatants were passed through a 0.22 μm filter and centrifuged at 4 °C for 3 h at 45,000×*g*. The viral pellet was re-suspended in 10 ml of phosphate buffered saline (PBS) and was stored at −80 °C as stock and used for experimental infection in mice and in vitro*.* The titer of the IAE-PR8 stocks was determined using a plaque titration assay, as described below. For viral inactivation, IAV-PR8 was boiled for 20 min and it was confirmed that there was no infectivity in the boiled viral solution by plaque titration assay.

### Animal experiment

All animal experiments were approved by the Animal Research Committee of NIBIOHN and Osaka University and all experiments were carried out following the Guidelines for the Japanese Animal Protection and Management Law, and with the approved standard operating procedure of the biosafety level facility.

Male C57BL/6 mice (CREA Japan Inc.) were randomly assigned into two groups; one group with live PR8 inoculation and another with boiled-inactivated PR8. PR8 inoculation experiments were performed as described previously with modifications [46]. To induce severe and acute brain edema mimicking IAE, 3- or 4-week-old mice were inoculated with several routes and several doses of PR8, from 1 × 10^4^ to 2 × 10^4^ median tissue culture infectious dose (TCID_50_) of PR8 intranasal inoculation, until the apparent neurological symptoms occurred 3 days post-inoculation [72 h post inoculation (hpi)]. Once the appropriate route and dose were fixed, the appropriate dose of the chosen route was modified according to the body weight of each mouse for further experiments, adjusting the variability in the body weight of developing young mice. After PR8 inoculation, body weight, neuromuscular function and neurological symptoms of either live or inactivated virus-inoculated mice were measured daily till 72 hpi. Neurological performance was assessed using clinical scoring of neuromuscular function [77]. Neurological symptoms were also assessed by observing seizures.

Mice were sacrificed under anesthesia at 3, 6, 24, 48, and 72 hpi to collect whole blood and brain tissue. Brain tissue was sectioned and weighted before use in each experiment. Evans blue dye (1%) was injected intravenously 1-h before sacrifice and perfused transcardially with PBS, for the vascular permeability study to confirm brain edema.

### Cell lines

HUEhT-1 cells (JCRB1458), U-251 MG cells (IFO50288) and MDCK cells (JCRB9029) were obtained from the JCRB Cell Bank of NIBIOHN. HUEhT-1 cells were grown in Molecular, Cellular, and Development Biology (MCDB) 131, including 10 mM of L-glutamine supplemented with 10% heat-inactivated fetal bovine serum (FBS), 30 μg/ml of endothelial cell growth supplement (ECGS), 5 μg/ml of heparin-Na on a collagen coated dish. U-251 MG was grown in Eagle’s minimum essential medium (EMEM) with 10% FBS. MDCK was grown in Dulbecco’s modified Eagle medium (DMEM) with 10% FBS and used mainly for viral proliferation and viral titration as mentioned at corresponding sections.

Cells with 90–95% confluency in 6-well plates were inoculated with PR8 at MOI = 1 (MOI = 0.01 for the viral quantitative assay) and cultured for 24, 48, and 72 h. The supernatant and inoculated cells were prepared to use for viral quantitative analysis, immunocytochemistry, real-time RT-PCR and Western blot, respectively.

### Human autopsy tissue

Human brain tissue samples were obtained from the Osaka General Medical Center and Osaka Women’s and Children’s Hospital. The brains of 3 patients with IAE (died in 1998–2004); CHARGE association as co-morbidity of a patient and tuberous sclerosis of another patient, and 1 control (died in 2014) were examined (Supplementary Table 1). Diagnoses of IAE with Reye’s syndrome or HSES were made by direct detecting of the IAV antigen and a typical clinical course for 2 cases. The diagnosis of IAE with Reye’s syndrome for 1 case was made by a typical clinical course and concurrence with an influenza epidemic. Autopsies were performed after obtaining written informed consents from the parents. This study was approved by the Central Ethics Review Boards of the Osaka University Hospital (23,194) for all facilities. This study was conducted following the Declaration of Helsinki and the Ethics Guidelines for Medical Research Involving Human Subjects.

Tissue obtained during the autopsy were immediately fixed in 10% formalin and processed into paraffin blocks. 5-μm-thick paraffin sections of the frontal cortex, temporal cortex, and cerebellum were used for immunohistochemical studies. The tissue was used after confirming that they did not contain tubers or areas of calcification.

### Antiviral drugs and chemicals

Baloxavir acid (BXA) was purchased from Shionogi Co. Ltd., and favipiravir (FVP) and peramivir trihydrate (PER) from Tokyo Chemical Industry. The drugs including cycloheximide (CHX) were prepared according to the manufacture’s data sheets and stored at −80 ℃. They were diluted with PBS upon use, and the concentration was adjusted with a series of dilutions for the LDH assay. 

For the intervention study of the mouse models, 4 mg/kg of BXA was subcutaneously injected [53] one day before and 8 h after viral inoculation, for pretreatment and 8 hpi treatment, respectively. 25 mg/kg of PER was also intramuscularly injected [41] one day before and 8 h after inoculation, as with BXA treatment (Fig. 7a).

### Cytotoxicity LDH assay

The LDH cytotoxicity release cell death assay was performed to analyze the effectiveness of antiviral drugs and chemicals for each cell line using the cytotoxicity LDH assay Kit-WST. The assay was performed following the manufacturer’s instructions. Adherent cells (MDCK cells, HUEhT-1 cells and U-251 MG cells) that were previously cultured for 3–4 days were grown to approximately 90% confluency. Each cell was washed, detached with trypsin, and pelleted. Cells were then counted using a Neubauer counting chamber, diluted with each cell culture medium, and seeded in 96-well plates at the number that reached 90% confluence at 24 h after seeding. 24 h later, the cell growth in the wells of the plates was confirmed confluency at 90–95% and the cell culture medium was removed, and the plates were washed thrice. 100 μl of VCM containing IAV-PR8 (MOI = 10), three-staged dilution series of each antiviral drugs/chemical (BXA, FVP, CHX, and PER) and controls were added to each well, and incubated for further 48 h in the incubator. A provided lysis buffer was added to the high control wells 30 min before the applications of the working solution, and then stop solution to all wells. The LDH absorbance released from each well was measured at 490 μm by a plate reader.

### Immunohistochemical/immunocytochemical analysis

Histological analyses of the animal model were performed as previously described [45]. In brief, the mice were anesthetized (isoflurane) and transcardially perfused with 4% paraformaldehyde (PFA) in PBS or saline. The brains were removed, equilibrated with 4% PFA/PBS, cryoprotected with 30% sucrose, and embedded in the OCT compound (Sakura Finetek) using 2-methylbutane cooled in liquid nitrogen. The brain tissue was then sectioned to an 8-μm thickness.

Histological analyses of the autopsy brains were performed with Hematoxylin and Eosin (H&E) staining following standard protocols. For immunohistochemistry, the brain sections of the animal model and autopsy were stained using the antibodies listed in the Supplementary Methods.

Areas of brain endothelial cells (EC) and IAV-nucleoprotein (NP)-stained areas from both live and inactivated group animals were calculated in pixels, using immunohistochemical lower magnification images (100×) of CD31 (EC) and IAV-NP. For the immunocytochemical analysis of inoculated cells, cells were washed with 1 × PBS and fixed with 10% formaldehyde/PBS for 30 min. After washed with 1 × PBS, cells were treated using the same procedures as in immunohistochemical studies.

All immunohistochemical slides were viewed on a Zeiss Axio Observer for bright field and fluorescence microscope (Carl Zeiss Co., Ltd). Dual or triple immunofluorescence slides were also imaged with Leica TCS SP8 Microscope capable of 3D (motorized XY stage and Z focus).

#### Viral quantitative analysis with animal brain tissue and infected cells

A plaque titration assay was performed as described previously [46]. Viral quantitative analysis of the animal brains, whole blood, and infected cells was performed according to the plaque titration assay with some modifications. For brain tissue and whole blood of mice, the collected tissue and whole blood were homogenized and sonicated in VCM, and the supernatant was titrated. Details were described in the Supplementary Methods.

#### Real-time RT-PCR

RNA from brain tissue and cells was purified using TRIzol ™ Reagent (Thermo Fisher Scientific) according to the manufacturer’s protocol. Viral RNA was quantified using the QuantiTect® Probe RT-PCR kit (QIAGEN). IAV-NP cycle threshold (Ct) value was analyzed using the Applied Biosystems 7900HT Fast Real-Time PCR system (Applied Biosystems Japan Ltd.) under the following conditions: 50 ℃ for 30 min., one cycle; 95 ℃ for 15 min., one cycle; 94 ℃ for 15 s, and 56 ℃ for 75 s, 45 cycles. The IAV RNA for the NP gene was detected using primers; a forward (5′-GARRTYATAARRATGATGG-3′), reverse (5′-ATTGTCTCCGAAGAAATAAG-3′), and TaqMan probes (5′-FAM-CGTCYGAGAGCTCRAARACTCCCC-MGB-3′) [65].

### Western blot analysis

The lysates including proteins from brain tissue and cells, were purified after the RNA and DNA were removed using TRIzol. The extracted lysates were dissolved in 1% SDS solution and sonicated. The supernatants of the lysates were harvested, loaded onto 10% SDS-PAGE gels (50 μg total protein per well) and transferred to a polyvinylidene difluoride membranes. The membranes were blocked with 5% dry milk for 1 h and incubated with an anti-IAV-NP antibody (flu A-NP; 1:1600, Gene Tex), anti-CD11b antibody (1:1000, Abcam), or an anti-β-actin antibody (1:500, Sigma Aldrich) in TBS-T (20 mM Tris–HCl pH7.6, 137 mM NaCl and 0.2% Tween 20®) at 4℃ overnight. Then, the membranes were washed with TBS-T three times each for 5 min and then incubated with horseradish peroxidase (HRP)-conjugated secondary-antibodies in TBS-T for 1 h at room temperature (RT). Protein signals were detected using a chemiluminescent substrate (Chemi-Lumi One, Nacalai Tesque Inc.) and the image was obtained using the ImageQunat™ LAS 500 (Cytiva).

### Statistics

A two-tailed Student’s *t-*test was used to determine the significance of the differences between the two groups, while one-way ANOVA was used for multiple different groups. The occurrence of death was assessed by Kaplan–Meier analysis and differences were determined using the log-rank test. Data are presented as mean ± SEM. Statistical significance was set at *P* < 0.05. GraphPad Prism 9.0 were used for statistical analyses and data visualization.

## Results

### Establishment of the Influenza virus-induced brain edema (IVE) model

**Fig. 1:**
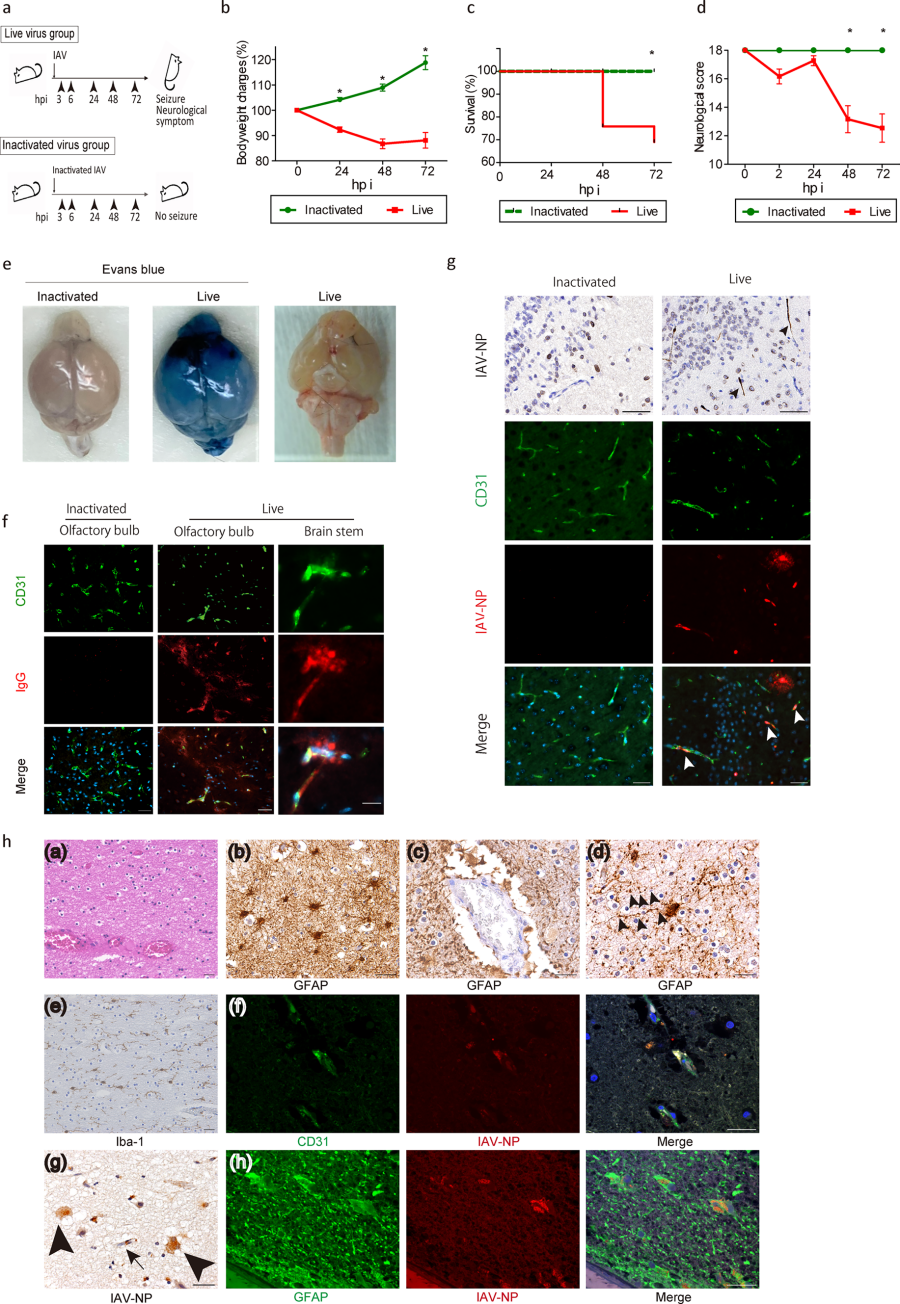
Massive deposits of viral protein in the brain of the Influenza virus-induced brain edema (IVE) models and the human IAE patients. **a** Protocol for inducing IVE. Three weeks-old male C57BL/6 mice were inoculated with Influenza A virus (IAV) A/PR/8/34 at a dose of 1.2 × 10^3^ TCID_50_/g. Mice were followed up at 3, 8, 24, 48, and 72 h post inoculation (hpi). Control mice were inoculated with inactivated IAV. **b** Change in body weight, **c** Kaplan–Meier analysis of survival rate, and **d** neurological scores of IVE mice, *n* = 36. **e** The brains of IVE mice harvested at 72 hpi with (*left and middle panels*) or (*right panel*) without injection with Evans blue dye. *n* = 3. **f** Representative images of brains from IVE mice harvested at 72 hpi stained with IgG. Red, IgG; *green*, CD31, a marker of endothelial cells (EC); *blue*, DAPI for nucleus counterstaining; *n* = 8. **g** Representative images of olfactory bulbs from IVE mice harvested at 72 hpi stained with anti-IAV nucleoprotein (NP). *Black and white arrowheads* indicate IAV-NP positive EC. Red, IAV-NP; *green*, CD31; *blue*, DAPI for nucleus counterstaining; *n* = 5. **h** Images of the autopsied brain from a patient with IAE. *(a)* Hematoxylin–Eosin (H&E) staining of the white matter of the frontal cortex, *(b, c, d)* immunohistochemical staining for GFAP of the cerebral white matter. *Arrowheads* indicate beaded astrocytic foot processes [*(d)*]. *(e)*immunohistochemical staining for Iba-1 of the cerebral white matter, *(f)* immunohistochemical staining of the cerebral cortex. *Red*, IAV-NP; green, CD31; *blue*, DAPI *(g)* immunohistochemical staining with anti-IAV-NP in the cerebral cortex. An *arrow* and *arrowheads* indicate endothelial cell and astrocytes, respectively. *(h)* immunohistochemistry of the cerebral cortex. *Red*, IAV-NP; *green*, a marker of glial fibrillary acidic protein (GFAP); *blue*, DAPI. *Scale bars* indicate [**f** *right panels*; **h ***(f)*, *(h)*] 20 μm, (**f** *left and middle panels*; **g**; **h** [*(a)*, *(b)*, *(c)*, *(d)*, *(e)*, *(g)*]) 50 μm. Data presented as mean ± SEM, *p < 0.05

An animal model mimicking IAE; IVE, was established in rodents by intravenous inoculation with live IAV at a dose of 1.2 × 10^3^ TCID_50_/g (Fig. 1a). IVE mice started to lose weight in 24 hpi and died within 48 hpi, while the control mice inoculated with inactivated IAV gained weight throughout the experimental period (Fig. 1b and c). Most of the IAE animals showed neurological symptoms, while the control mice were asymptomatic (Fig. 1d). Seizures and death were observed in 24 and 31%, respectively within 72  hpi in the IVE mice (Supplementary Fig. 1A). Inconsistent increases in inflammatory cytokines in serum were observed in the IVE mice, even in cases without apparent symptoms, while inflammatory cytokines also increased in some control mice (Supplementary Fig. 1B). Immunohistological and Western blot analyses confirmed the lack of microglial proliferation and the decreased microglial protein levels during the observational period (Supplementary Fig. 1C and 1D), as reported in IAE patients [44, 67].

The brains of the IVE mice showed diffuse leakage of Evans blue dye, while the control brains did not (Fig. 1e). The brains of IVE mice also showed bleeding or petechial hemorrhage in the olfactory bulb, cortex, brain stem, and cerebellum (Fig. 1e). Immunohistochemistry revealed that IgG leaked around the EC both in the olfactory bulb and brain stem, with no leakage in the brain of the inactivated animals (Fig. 1f). Overall, the intravenous inoculation of live IAV induced IVE, whose pathological characteristics were quite similar to those of IAE.

### Massive deposits of viral protein in brain EC of the IVE mice and the human IAE patients

To investigate the involvement of viral dynamics in the formation of IAE pathogenesis, we first examined the presence of viral proteins in the brains. The brains of the IVE mice revealed the presence of massive viral NP deposition in the brains as shown in immunohistochemistry (Fig. 1g). Viral protein deposition is mostly present in EC colocalized with CD31. These viral deposits were scattered around almost all areas of the brain, especially in the olfactory bulb and thalamus, while viral protein was absent in the brains of mice inoculated with inactivated virus (Fig. 1g).

**Fig. 2:**
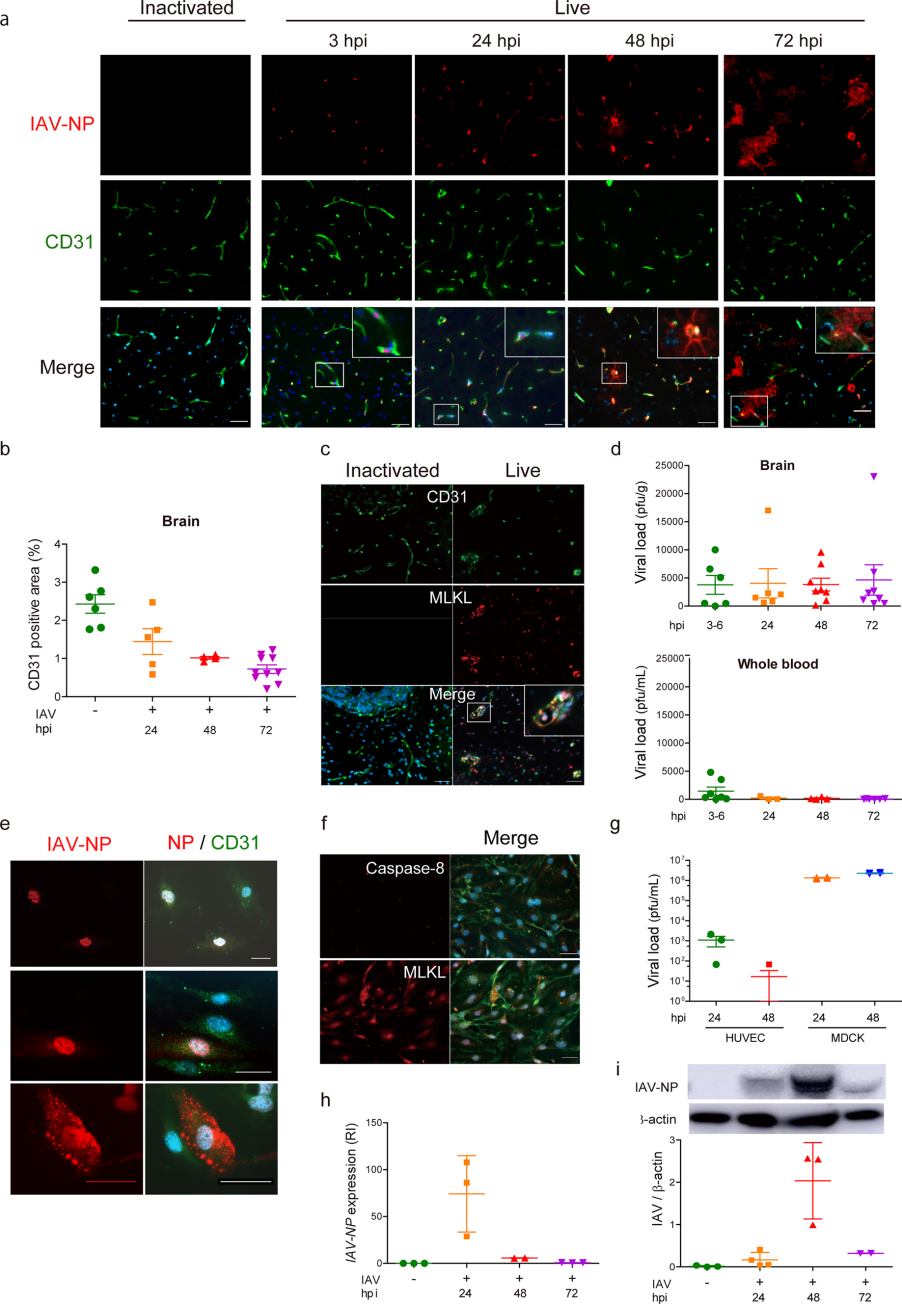
IAV invades brain endothelial cells and induces necrosis. **a** Representative images of the thalamus of IVE mice harvested at the indicated hpi stained with anti-IAV-NP antibody. *Red*, IAV-NP; *green*, CD31; *blue*, DAPI, *n* = 3. **b** CD31-positive area of brains, *n* = 4–10 **c** Images of the brains of IVE mice at 72 hpi stained with an anti-phospho-MLKL antibody. *Red*, an anti phospho-MLKL (a marker of necroptosis); *green*, CD31; *blue*, DAPI, *n* = 3. **d** Plaque titration assay of the brain (*upper panel*) and whole blood (*lower panel*) harvested from IVE mice at indicated hpi, *n* = 6–8. **e** Immunohistochemical analysis of HUVEC at 24 hpi stained with an anti-IAV-NP antibody. *Red*, an anti-IAV-NP; *green*, CD31; *blue*, DAPI, *n* = 6. **f** Immunohistochemical analysis of HUVEC at 24 hpi stained with an anti-caspase-8 or an anti-MLKL antibody. *Red*, caspase-8 (a marker of apoptosis) (*upper panel*); MIKL (a marker of necroptosis) (*lower panel*); *green*, CD31; *blue*, DAPI, *n* = 3. **g** Plaque titration assay of HUVEC or MDCK cells at indicated hpi, *n* = 3–4. **h** Expression of the IAV-*NP* gene and **i** quantification of IAV-NP by western blotting in HUVEC at the indicated hpi, RI, relative index, *n* = 3–4. *Scale bars* indicate (**a**, **c**, **e**, **f**) 50 μm. Data presented as mean ± SEM, *p < 0.05

**Fig. 3:**
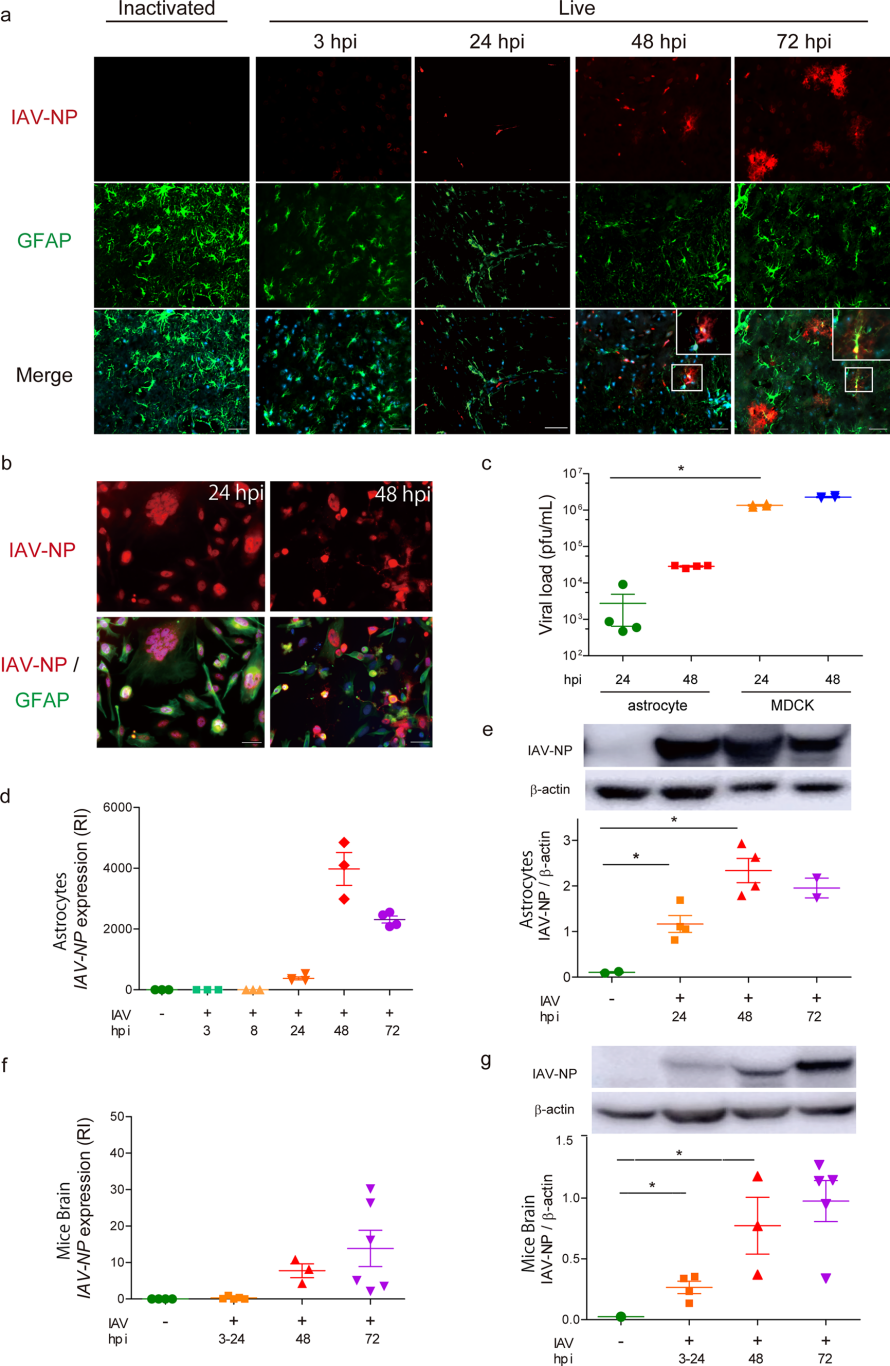
Infection of IAV in EC precedes viral protein depositions in astrocytes. **a** Representative images of brains (thalamus, olfactory bulb and cerebellum) from IVE mice harvested at the indicated hpi stained with anti-IAV-NP and anti-GFAP antibody. *Green*, GFAP (a marker for astrocytes); *red*; IAV-NP; *blue*; DAPI, *n* = 5. **b** Images of cultured astrocytes in 24 (*left panels*) and 48 (*right panels*) hpi stained with an anti-IAV-NP and an anti-GFAP antibodies. *Green*, GFAP; *red*, IAV-NP; *blue*, DAPI, *n* = 4. **c** Plaque titration assay of cultured astrocytes and MDCK cells at indicated hpi. *n* = 24. **d** Expression of IAV-*NP* gene and **e** quantification of IAV-NP in cultured astrocytes at the indicated hpi, RI, relative index, *n* = 4. **f** Expression of IAV-*NP* gene and **g** quantification of IAV-NP in the brains of IVE mice at the indicated hpi, RI, relative index, *n* = 3–7. *Scale bars* indicate **a**, **b** 50 μm. Data presented by mean ± SEM, *p < 0.05

We confirmed these findings with the analysis of the human autopsy brain tissue. The autopsied brains of patients with IAE showed characteristic histological changes as follows: (1) diffuse brain edema with spongiosis and perivascular exudates especially in the white matter (Fig. 1h (a)), (2) bleeding, congestion and astrogliosis composed of hypertrophic and glial fibrillary acidic protein (GFAP)-immunoreactive astrocytes (Fig. 1h (b)), (3) astrocytic end feet were irregularly interrupted in the perivascular blood brain unit (Fig. 1h (c)) and many hypertrophic astrocytes showed beading and fragmentation of the astrocytic processes (clasmatodendrosis) (Fig. 1h (d): arrowheads), (4) ramified microglia, immunoreactive for Iba-1 showed no marked increase in the white matter (Fig. 1h (e)), and (5) the IAV-NP-immunoreactivity was observed in the brain blood vessels and astrocytes, showing colocalization with viral proteins in the IAE autopsied brain (Fig. 1h (f)(g)(h), g: an arrow for EC and arrowheads for astrocytes).

### IAV invaded brain EC and induced necrosis.

We investigated the viral dynamics in the brains of IVE mice. After blood-borne inoculation of IAV, the IAV protein was first localized in the brain EC of the IVE mice. IAV-NP was first localized in the EC nuclei at 3 hpi, gradually spread into the EC cytosol by 24–48 hpi with rather rough and smaller appearance of EC itself, and spread outside the EC at 48–72 hpi (Fig. 2a). At the same time, EC gradually decreased over time with disruption, halving at 72 hpi (Fig. 2b). The infected EC tended to undergo necrosis, as stained with a necroptosis marker, MLKL, but not an apoptosis marker, Caspase-8 (Fig. 2c and Supplementary Fig. 2A). The plaque titration assay of IVE mice showed the continuous detection of live virus in brain tissue from 3 to 72 hpi, despite its quick disappearance from the whole blood (Fig. 2d). IAV usually infects by the airway route, and we tested whether intranasal inoculation caused a similar pathology in brain. We also detected the IAV antigen from the brain EC of IAV intranasally inoculated mice, although it was less efficient (Supplementary Fig. 2B). This way, IAV could reach the brain EC hematogenously in mice, even in case of intranasal inoculation.

Next, we inoculated IAV into HUEhT-1, an EC cell line, to confirm the virulence of IAV to EC. IAV-NP was detected in the EC nuclei with occasional deposit-like dots in the cytoplasm at 24 hpi after IAV inoculation (Fig. 2e). EC at 48 hpi was stained with an anti-MLKL antibody, but not with an anti-caspase-8 antibody (Fig. 2f). This seemed to be a unique cell death observed in EC inoculated with IAV, since IAV-infected MDCK or type 2 alveolar epithelial cells died through apoptosis and necroptosis [70]. IAV scarcely amplified in the cultured EC according to the viral titration assay compared with MDCK cells, which maintained a high viral load even after 48 hpi (Fig. 2g). Expression of IAV-*NP* RNA peaked at 24 hpi followed by a rapid decrease after 48 hpi (Fig. 2h). In contrast, the protein peaked at 48 hpi (Fig. 2i).

In summary, IAV infected EC but did not amplify well, induced necroptosis, and its protein accumulated in EC in vitro. Despite less proliferation, viral protein accumulation seemed to be a notable feature of IAV infection in EC.

### IAV infection to EC preceded viral protein depositions in the model brains

In addition to EC, we next focused on astrocytes, since the IAV antigen was detected in them. In Fig. 2a, IAV-NP looked like to exist outside EC at 48–72 hpi. This could be explained as follows; IAV-NP might be located outside EC cell bodies because of EC disruption with IAV infection which was shown in vitro study and spread outside EC and other cells including astrocytes shown in Fig. 3a. NP was detected in the cytosol of astrocytes at and after 48 hpi (Fig. 3a). Along with spread of the IAV-NP stainable area, GFAP signal was weakened with time. Next, we inoculated IAV in U-251 MG cells, an astrocyte cell line, to investigate the virulence of IAV in astrocytes. IAV-NP was detected in astrocyte nuclei with a cytopathic effect (CPE) of syncytium 24 hpi after IAV inoculation. At 48 hpi, IAV-NP was detected in astrocyte cell body (Fig. 3b). IAV proliferated in cultured astrocytes, although the amount was much lower than that in MDCK cells (Fig. 3c). The expression of IAV-*NP* RNA increased after 48 hpi (Fig. 3d), and the protein level of IAV-NP increased until 48 hpi (Fig. 3e). Unlike IAV dynamics in cultured EC, in which the viral protein only showed a peculiar behavior compared to viral amplification, viral amplification, viral protein NP expression, and viral RNA expression of IAV-*NP* showed the same tendency in cultured astrocytes.

Next, we profiled the protein and RNA levels of IAV in the brains of IVE mice. The expression of IAV-*NP* RNA in IVE brains increased over time, although the change was moderate (Fig. 3f). Western blot analysis of IAV-NP in IVE brains revealed that the NP protein increased with time until 72 hpi (Fig. 3g).

In summary, IAV entered the brain EC and gradually spread outside EC including astrocytes. IAV proteins accumulated in the brains at 48–72 hpi without an apparent increase of live virus and viral RNA in IVE mice, suggesting that this protein accumulation should be related to IAE progression.

#### IAV protein deposited in the brain EC of the IAE patients

**Fig. 4:**
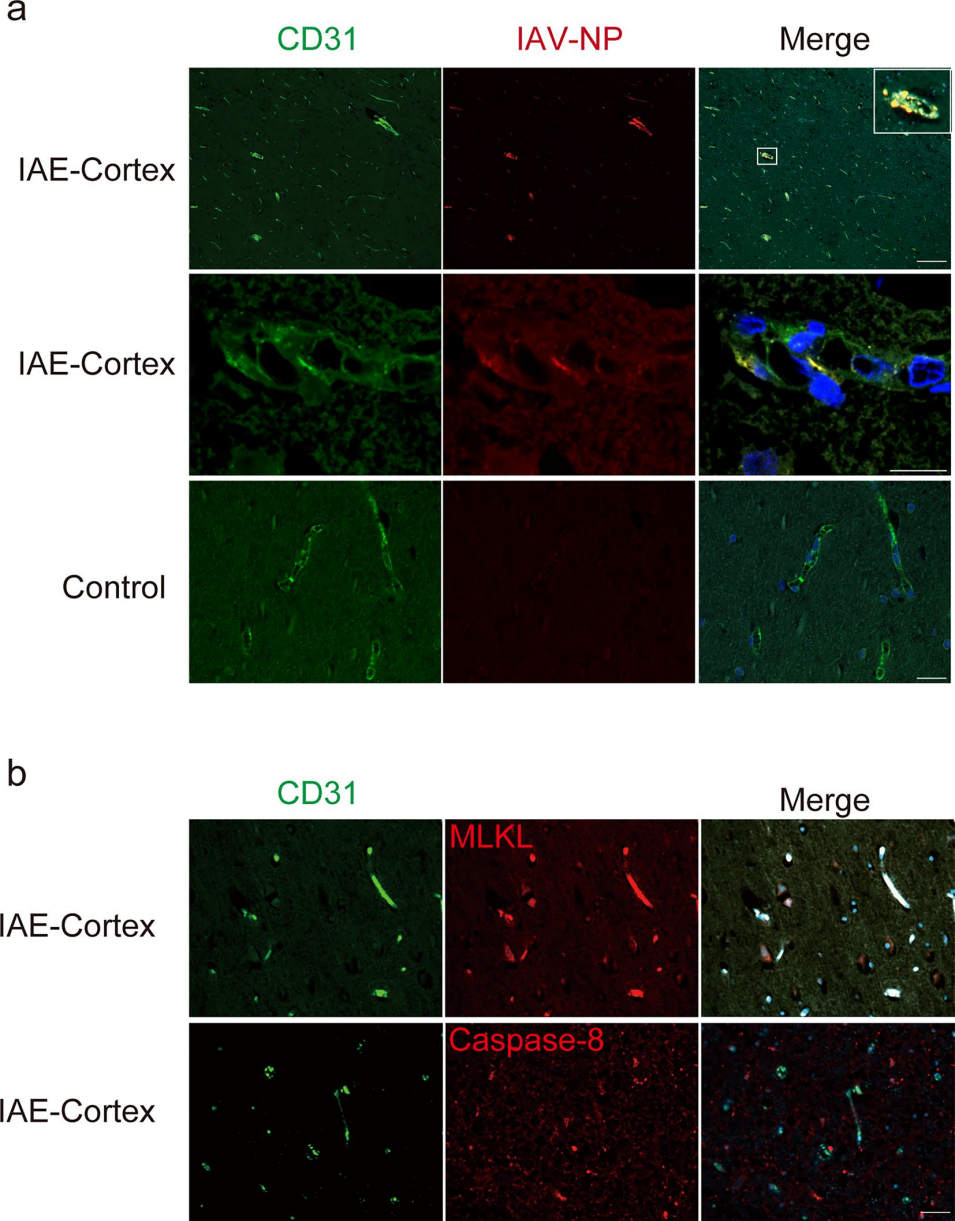
IAV-NP depositions in the brain EC and astrocytes of the IAE patient. **a** Upper panels: a lower magnification view (×100) of the cortex from the brain of IAE patients, *middle and lower panels*: a higher magnification view (×400) of the cortex from the brains of the IAE (*middle*) and a control (*lower*) patient. *Green*, CD31; *red*, IAV-NP; *blue*, DAPI. **b** Staining of the cortex from the brain of the IAE patients. *Green*, CD31; *red*, (*upper panels*) MLKL; or (*lower panels*) caspase-8; *blue*, DAPI. *Scale bars* indicate 50 μm (**a** *middle and lower panels*, **b**) and 400 μm (**a** *upper panels*)

We next investigated whether the accumulation of viral protein was present in the brains of the patients with IAE. Analysis of the autopsied brains of the IAE patients showed that IAV-NP was localized to several brain areas. IAV-NP was present in the nuclei and cytosol of EC in the brains of IAE patients, but no viral protein in a control brain (Fig. 4a). Most brain EC expressed cell death signals and colocalized with MLKL, but not with apoptosis marker, the latter of which was expressed in cells other than brain EC (Fig. 4b).

In summary, antigen of viral protein was present in EC of the human autopsy brains, as with the case in the brains of the IVE mice.

#### Endothelial leakage and fragmentation of the astrocytic foot process were observed in IVE mice and the human IAE patients

Although the lack of AQP4 ameliorates cytotoxic brain edema, AQP4 exacerbates vasogenic edema observed in IAE and other diseases with blood brain barrier (BBB) disruption [56, 76]. Fragmentation of astrocytic foot processes (clasmatodendrosis) was observed in brains of patients with IAE and many other acute and chronic neurological conditions [4, 25, 86]. We examined the temporal relationship between fragmented foot processes and AQP4 in IVE mice and in the IAE patients.

**Fig. 5:**
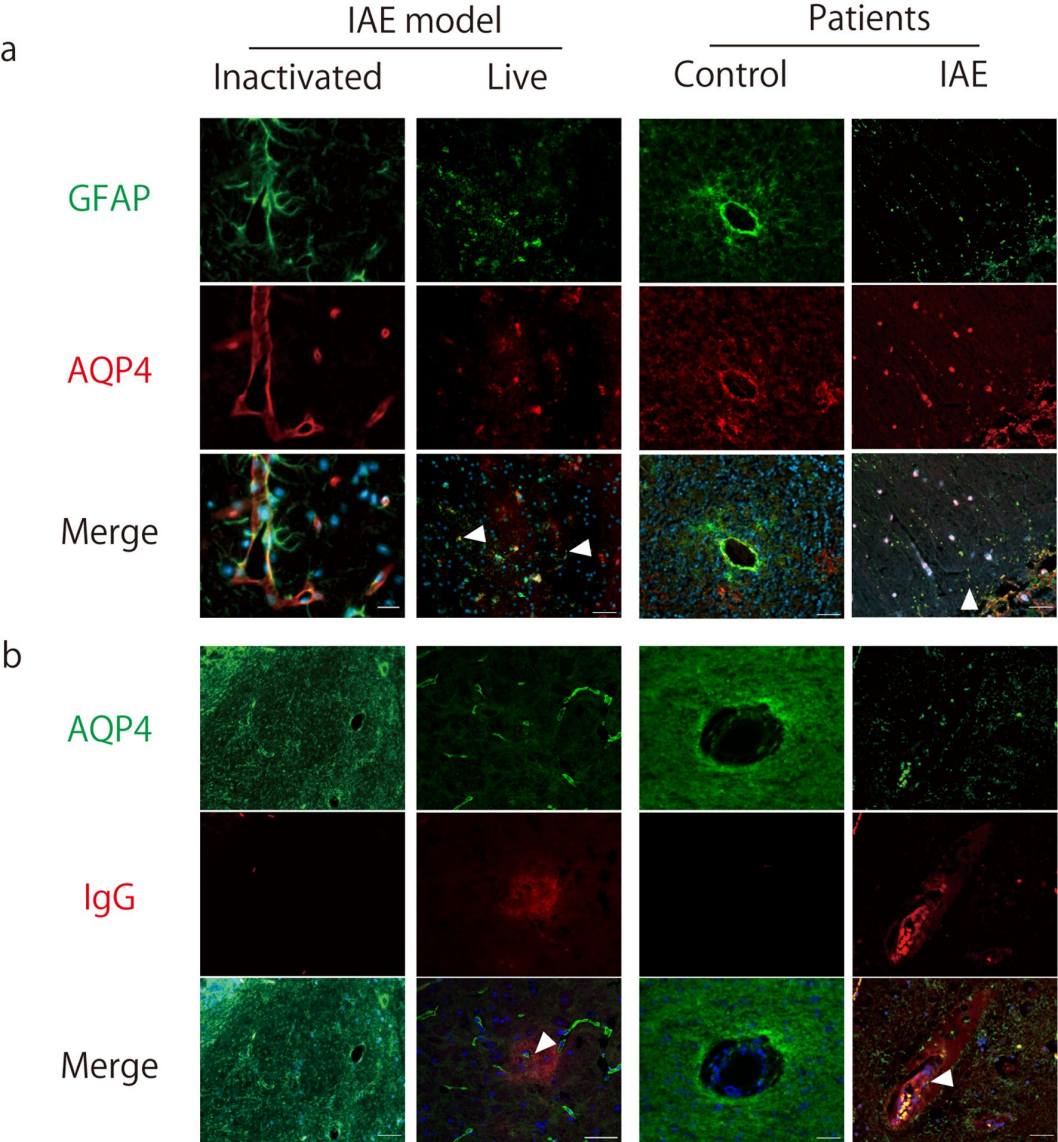
Endothelial leakage and fragmentation of the astrocytic foot process are observed in the brains of IVE mice and in the human IAE patients. **a**, **b** Representative images from the brains of mice inoculated with inactivated IAV (*left panels*), IVE mice harvested at 72 hpi (live, *left middle panels*), a human control (*right middle panels*), and patients with IAE (*right panels*). **a** *Green*, GFAP; *red*; aquaporin 4 (AQP4); *blue*, DAPI. *Arrowheads* indicate fragmented astrocytic processes. **b** *Green*, AQP4; *red*, IgG; *blue*, DAPI. *Arrowheads* indicate AQP4-positive fragmented foot process in the IgG leaked area. *Scale bars* indicate 50 μm

In mouse brains inoculated with inactivated IAV, GFAP stained cell bodies and foot processes of astrocytes, while AQP4 stained astrocytes foot processes mainly in the perivascular area. In the brains of IVE mice, the colocalizing area moved to fragmentated astrocytic foot processes, which was also observed in the IAE patient brains (Fig. 5a, arrowheads). The AQP4-positive fragmented foot processes were also observed in the IgG leaked area in the brains of the IVE mice and IAE patients (Fig. 5b, arrowheads).

#### Inhibition of viral transcription and/or translation suppressed the endothelial cell death

**Fig. 6:**
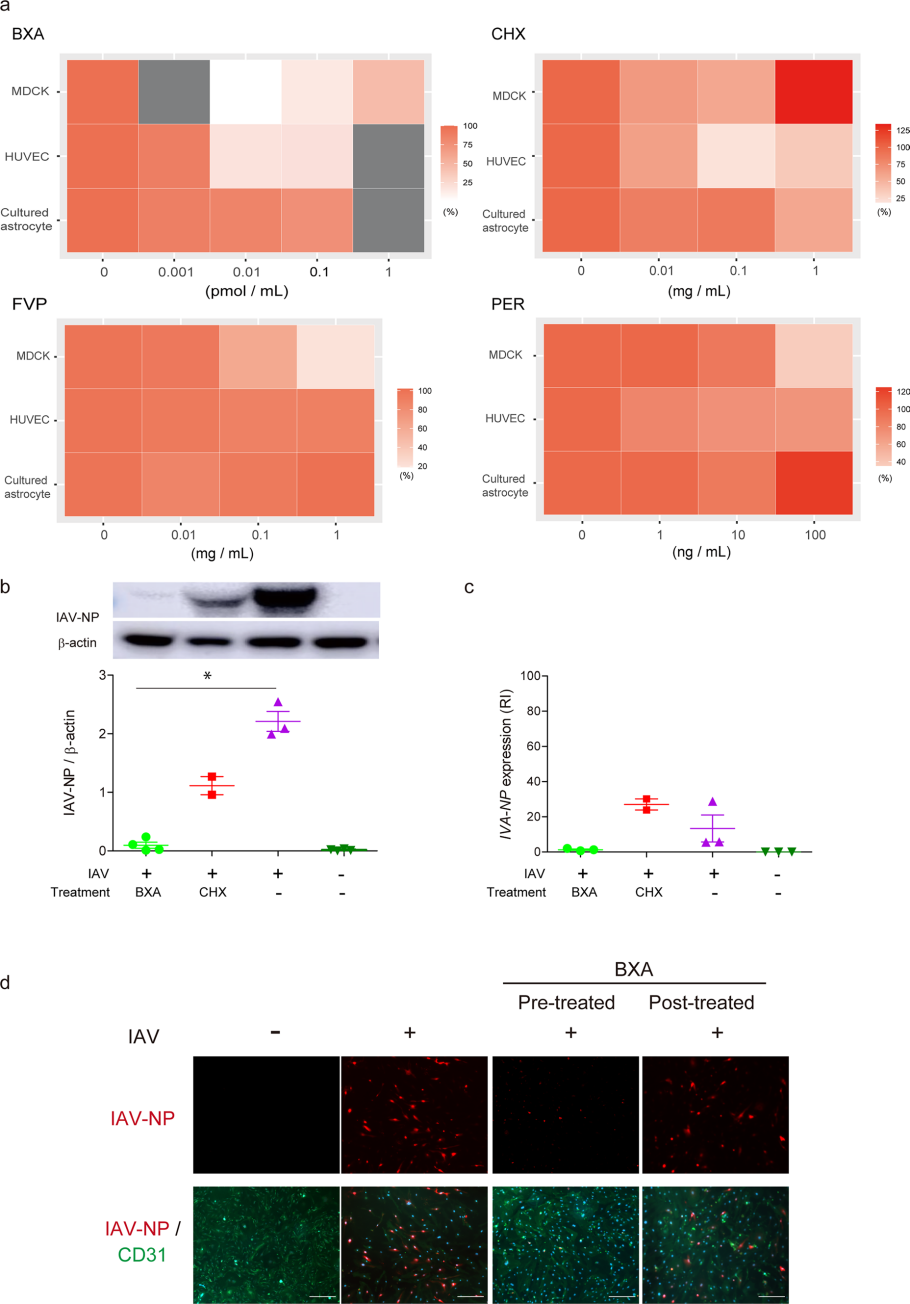
Inhibitions of viral transcription and/or translation suppresses endothelial cell death. **a** The cytotoxicity of IAV-inoculated MDCK, HUVEC and cultured astrocytes treated with Baloxavir acid (BXA), favipiravir (FVP), cycloheximide (CHX), and peramivir trihydrate (PER). Results are shown in heatmap display. *Gray box*, not tested.* n* = 3. **b** Quantification of IAV-NP. **c** Expression of the IAV-*NP* gene in IAV-inoculated HUVEC treated with BXA or CHX. RI, relative index; *n* = 2–3. **d** Images of IAV-inoculated HUVEC at 48 hpi pre- or post-treated with BXA. Immunocytochemical staining was performed using an anti-IAV-NP and an anti-CD31 antibodies; *n* = 2–3. *Green*, CD31; *red*, IAV-NP; *blue*, DAPI. *Scale bars* indicate 50 μm

Our data showed that the entry of virus into EC followed by astrocytic damage was thought to trigger the onset of IAE. Despite the absence of viral proliferation, the accumulation of IAV-NP in cultured EC suggested that IAV proteins, but not viral proliferation, should damage EC. To test this hypothesis, we treated IAV-inoculated EC and related cells with known anti-influenza drugs and the protein translational inhibitor, CHX. BXA, an inhibitor of cap-dependent endonuclease for influenza viral mRNA synthesis, reduced cell death rates by 80–90% in MDCK and HUVEC, while BXA was ineffective in cultured astrocytes (Fig. 6a-BXA). CHX also rescued the cell death in HUVEC, while the effects of CHX were limited to half in MDCK cells and cultured astrocytes (Fig. 6a-CHX). FVP, an inhibitor of influenza virus RNA-dependent RNA polymerase, and PER, a neuraminidase inhibitor, were effective against MDCK cells, but not against the other two cell lines (Fig. 6a-FVP, PER). Blockade of viral mRNA synthesis or protein translation suppressed IAV-related cell death in HUVEC, while the protective effects of the tested drugs were limited in cultured astrocytes.

Next, we examined the viral protein and RNA levels of IAV-inoculated EC, treated with BXA and CHX. The level of IAV-*NP* was markedly reduced by BXA, and reduced by half by CHX in IAV-inoculated HUVEC compared to that of the IAV-inoculated control without treatment (Fig. 6b). BXA also suppressed the expression of IAV-*NP* RNA in IAV-inoculated HUVEC, while CHX did not (Fig. 6c). BXA almost completely blocked the RNA expression and protein synthesis of IAV-NP in HUVEC, while CHX reduced the viral protein but not the viral RNA. Thus, blockade of viral transcription and translation could be the key for protecting EC from IAV.

Since the protective effect of BXA against IAV in HUVEC was almost complete, we further examined whether treatment with BXA was still effective in HUVEC after IAV inoculation. As a result, post-treatment with BXA at 8 hpi partially decreased the number of NP-positive HUVEC (Fig. 6d).

#### Inhibition of viral transcription and translation prominently improved lethality of IVE mice

**Fig. 7:**
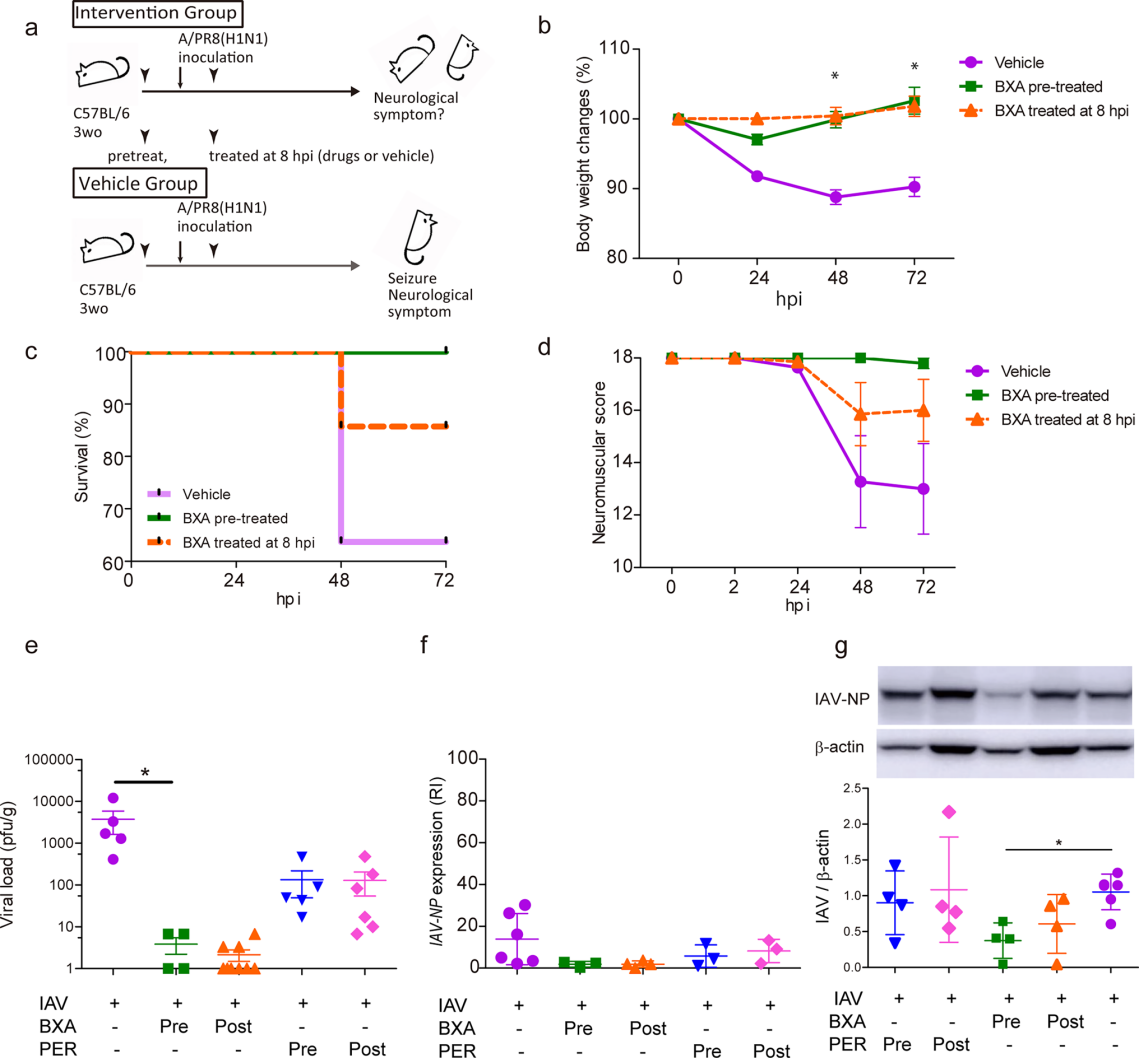
Prominent suppression of lethality in IVE mice by inhibiting viral transcription and translation. **a** Schematic diagram of the experimental schedule. Male IVE mice were treated with BXA, either before (pre-treatment) or 8 h after inoculation (post-treatment). **b** Change in body weight, **c** Kaplan–Meier analysis of the survival rate, and **d** neurological scores of IVE mice; *n* = 5–11. **e** Plaque titration assay of the brains of IVE mice treated with BXA or PER. *n* = 4–9. **f** Expression of the IAV-*NP* gene and **g** quantification of IAV-NP in brains of IVE mice at 72 hpi treated with BXA or PER. RI, relative index; *n* = 3–6. *Red*, IAV-NP; *green*, CD31; *blue*, DAPI; *n* = 3. *Scale bars* indicate 50 μm. Data presented as means ± SEM, *p < 0.05

Finally, we investigated the effects of BXA in the IVE mice. The mice were treated with BXA before or after IAV inoculation (Fig. 7a). BXA treatment, both pre-treatment and post-treatment, suppressed reduction in body weight (Fig. 7b). Kaplan–Meier analysis showed complete survival in BXA pre-treated mice and a 50% reduction in lethality in BXA post-treated mice (Fig. 7c). Analysis of neuromuscular scores revealed that BXA pre-treated mice showed almost no neurological symptoms at 72 hpi, and the scores were improved by half in BXA post-treated mice (Fig. 7d). We also tested the effects of PER in the IVE mice (Supplementary Fig. 3A–D). Only pretreatment with PER improved survival rates and neuromuscular scores.

The plaque titration assay of animal brains showed almost no live IAV detection in BXA-treated mice and decently reduced viral load in PER-treated mice compared to vehicle-treated mice (Fig. 7e). The expression of IAV-*NP* RNA was lower, although without significance, in the brains of BXA- and PER-treated mice than in vehicle-treated mice (Fig. 7f). The protein level of IAV-NP in the brains was lower only in BXA-treated mice when BXA was pre-treated (Fig. 7g). In contrast, treatment with PER did not affect IAV-NP protein levels in the brains of IVE mice. Throughout the interventional experiments, protein levels of IAV-NP, but not viral load or IAV-*NP* RNA expression, correlated with the lethality and physical scores of the IVE mice. Post-treatment with BXA partially reduced the IAV-NP synthesis and lethality and suppressed the viral load and RNA expression, confirming the importance of viral transcription and translation in IAE pathogenesis. Pre-treatment with BXA completely improved the lethality of the IVE mice by inhibiting viral transcription and translation.

## Discussion

This study showed that IAV invaded the brain from brain EC and that viral proteins accumulated mainly in brain EC, resulting in brain edema or equivalently IAE using the IVE mouse model. The influenza virus was proved to enter the brain EC in IVE mice and patients with IAE. Although IAV did not show prominent proliferation in mouse brains, IAV-NP accumulated in the brains of the IVE mice and the IAE patients and spread from EC to outside parenchyma or cells including astrocytes. The accumulation of viral protein should be responsible for the severe clinical course and lethal prognosis in IVE mice. Consistent with this, an antiviral drug that blocked viral transcription and translation showed the complete effects in the treatment of IVE mice.

Several IAE related animal models have been reported as mentioned: encephalitis like models using none-seasonal influenza virus, animal models using KO mice and animal models using drugs which act to exaggerate brain edema [5, 10, 36, 55, 60, 66, 80, 90, 93, 95, 98, 101]. Among these models, ‘mouse models for Reye’s syndrome’ and the suckling period mouse models using juvenile visceral steatosis (JVS) mice had presented severe brain edema by inoculating epidemic influenza virus without adding any other drugs, as well as our IVE mice [80, 101]. Both models showed influenza virus antigen from brain endothelial cells, although the concise viral dynamics were unknown. Here we partially showed the viral dynamics and the possibility of treatment in IAE pathophysiology with IVE models and IAE autopsy brains.

The IVE model mice recapitulated many salient characteristics of human IAE: an acute and aggressive course, brain edema, increased inflammatory cytokines observed in two-thirds of animals, and typical pathological features; leakage of plasma components to the brain parenchyma, bleeding/hemorrhage, lack or least accumulation of inflammatory cells, and astrocytic clasmatodendrosis. Increased inflammatory cytokines was very variable in IVE mice, which was also observed in patients with IAE [26, 62], indicating that the increase in cytokines in IAE would be one of sufficient conditions causing the disease. These characteristics were especially observed in ANE, HSES, or other severe types of IAE.

The detection of influenza viral antigen in the brain parenchyma of IVE mice and patients with IAE was a key finding of this study. In the present study, we detected live virus and viral RNA in the IVE mouse brains and viral protein accumulation in the brains of both the IVE mice and IAE patients. Previous studies have also reported the viral detection from neurons, EC, astrocytes, and CD8-positive cells in IAE autopsy brains [19, 23, 37, 88, 99]. The routes to the extra-respiratory organs, especially central nervous system (CNS), of influenza virus invasion has long been a debate [49]. Although hematogenous spreading is a popular route, the low amounts and frequencies of viral antigens detected in the CNS or other extra-pulmonary organs are the weak points of this hematogenous route theory. Although our present IVE mouse model was established based on this hematogenous route, mice with intranasal inoculation also showed IAV antigen in the brains without apparent neurological symptoms, though the level was low (Supplementary Fig. 2B). Several studies have reported that IAV (H5N1) live virus, antigen, or RNA was detected in serum or autopsied brain [9, 82], and detection of IAV RNA was also reported in the 2009 pandemic (H1N1) and IAV (H3N2) infection [17, 75]. These detection of the viral antigen in brain EC and in vasculature were also reported with intranasally inoculated mice [13, 59].

The present study detected IAV live virus, viral RNA and viral protein in the brains of IVE model mice. In IVE mice, live virus disappeared from the vasculature within 24 h after IAV intravenous inoculation and live virus in the brains did not increase with time. These results indicated that IAV proliferated neither in the blood nor in the brains in IVE mice, and therefore, the viral loads were scarce in IVE mice brains. This is consistent with the paucity of viral antigens detected in the brains of patients with IAE. Despite the paucity of viruses in the IVE brains, viral existence in the brain itself seems pathological. In cultured EC, IAV decreased the number of daughter virus less than one tenth (Fig. 2g) consistent with earlier studies [29, 84]. In another cultured EC, IAV has been reported to enter human lung microvascular endothelial cells (HMVEC) and promote vascular leakage without apparent proliferation [2, 24, 68]. As seen in vitro and in vivo, IAV caused denature of EC without viral proliferation. After denaturing EC, IAV spreads to the brain parenchyma and a decrease of brain EC and increased vascular permeability led to brain edema. In this way, the temporal and spatial shifts of brain lesions should be linked to the onset and formation of IAE. Given these perspectives, unlike the IAV respiratory infection via airway epithelial cells, IAV seems to enter brain via EC and causes severe pathogenesis without regular proliferation. How viral protein spreads from EC to brain parenchyma and other CNS cells is yet to be solved.

Our data suggests that IAV should induce severe brain lesions despite less proliferative characteristics. We observed the key discrepancy between the amount of IAV protein and viral load. The amounts of viral protein NP increased over time, while the viral loads did not. Based on these observations, the direct pathogenesis of IAV proteins in IAE was tested using anti-influenza drugs in the present study.

Anti-influenza drugs were effective in MDCK cells in vitro. In contrast, only drugs that inhibit transcription and/or translation were effective in HUVEC, and all drugs tested showed little or no effect in cultured astrocytes. Notably, CHX; an inhibitor of translation, was highly effective in HUVEC, with only limited effects in MDCK cells. This indicates that HUVEC cell death can be potentially explained by synthesis of viral proteins rather than by viral proliferation. This was evident compared to the mechanisms of antiviral drugs in MDCK cells, in which any blockade of the IAV proliferation cascades was effective. Drugs that were effective in HUVEC in vitro were also effective in the IVE mice, suggesting that inhibition of transcription/translation, but not viral replication or proliferation, could prevent the progression of IAE pathogenesis. IAV virulence in brain EC was revealed to be the most important in IAE pathogenesis, since BXA was effective in IVE mice despite the limited effects in astrocytes in vitro.

Although influenza virus infects EC or astrocytes [29, 97], anti-influenza drugs has rarely been tested in these cells. The mechanisms of action of anti-influenza drugs are variable [21, 48, 85], and it is important which drug to be selected for each cell line. In fact, neuraminidase inhibitors and RNA-dependent RNA polymerase (RdRp) inhibitors were effective for MDCK, an epithelial cell line. However, these drugs had a limited effect on EC and IVE mice. The discrepancy of the trigger or entry point, between the IAE and the regular respiratory influenza infections, was responsible for the difference in the efficacy of antiviral drugs. Our study showed the importance of identifying a key process of each IAV-related pathogenesis and that different pathogeneses led to the different efficacies of drugs.

Despite propensity to induce viral resistance [74], only BXA is currently available as a drug that inhibits viral transcription and/or translation. Even under the restricted conditions, combined use of BXA and other anti-influenza drugs should be effective for IAE while reducing the risk of viral resistance.

This study showed the temporal-spatial dynamics of IAV in the pathogenesis of IAE. IAV entry into brain EC triggers the pathogenesis of IAE and promotes the accumulation of IAV protein in EC and brain parenchyma without apparent viral proliferation. The resultant denaturation of EC followed by astrocytic damage leads to brain edema and severe neurological symptoms in IVE mice. Inhibitors of viral transcription and/or translation are effective in rescuing EC denaturation and improving the lethality of IAE models.

The IAE pathogenesis, with severe brain edema caused by IAV, could be established without viral proliferation. The regulation of viral transcription and translation is the key to treatment of IAE.

### Supplementary Information

Below is the link to the electronic supplementary material.

Supplementary file1 (PDF 384 KB)

## Data Availability

The authors confirm that the data supporting the findings of this study are available within the article and its supplementary materials.

## References

[1] Amin R, Ford-Jones E, Richardson SE, MacGregor D, Tellier R, Heurter H (2008). Acute childhood encephalitis and encephalopathy associated with influenza: a prospective 11-year review. Pediatr Infect Dis J.

[2] Armstrong SM, Wang C, Tigdi J, Si X, Dumpit C, Charles S (2012). Influenza infects lung microvascular endothelium leading to microvascular leak: role of apoptosis and claudin-5. PLoS ONE.

[3] Azuma J, Nabatame S, Nakano S, Iwatani Y, Kitai Y, Tominaga K (2015). Prognostic factors for acute encephalopathy with bright tree appearance. Brain Dev.

[4] Balaban D, Miyawaki EK, Bhattacharyya S, Torre M (2021) The phenomenon of clasmatodendrosis. Heliyon 7(7):e07605. 10.1016/j.heliyon.2021.e07605 10.1016/j.heliyon.2021.e07605 PMC8326353 34368479

[5] Bissel SJ, Giles BM, Wang G, Olevian DC, Ross TM, Wiley CA (2012). Acute murine H5N1 influenza A encephalitis. Brain Pathol.

[6] Britton PN, Blyth CC, Macartney K, Dale RC, Li-Kim-Moy J, Khandaker G (2017). The spectrum and burden of influenza-associated neurological disease in children: combined encephalitis and influenza sentinel site surveillance from Australia, 2013–2015. Clin Infect Dis.

[7] Chen Y, Mizuguchi H, Yao D, Ide M, Kuroda Y, Shigematsu Y (2005). Thermolabile phenotype of carnitine palmitoyltransferase II variations as a predisposing factor for influenza-associated encephalopathy. FEBS Lett.

[8] Chen LW, Teng CK, Tsai YS, Wang JN, Tu YF, Shen CF (2018). Influenza-associated neurological complications during 2014–2017 in Taiwan. Brain Dev.

[9] Chutinimitkul S, Bhattarakosol P, Srisuratanon S, Eiamudomkan A, Kongsomboon K, Damrongwatanapokin S (2006). H5N1 influenza A virus and infected human plasma. Emerg Infect Dis.

[10] Cisse Y, Wang S, Inoue I, Kido H (2010). Rat model of influenza-associated encephalopathy (IAE): studies of electroencephalogram (EEG) in vivo. Neuroscience.

[11] Cleuziou P, Renaldo F, Renolleau S, Javouhey E, Tissieres P, Leger PL (2021). Mortality and neurologic sequelae in influenza-associated encephalopathy: retrospective multicenter PICU cohort in France. Pediatr Crit Care Med.

[12] Davis LE, Blisard KS, Kornfeld M (1990). The influenza B virus mouse model of Reye’s syndrome: clinical, virologic and morphologic studies of the encephalopathy. J Neurol Sci.

[13] Davis LE, Kornfeld M, Daniels RS, Skehel JJ (2000). Experimental influenza causes a non-permissive viral infection of brain, liver and muscle. J Neurovirol.

[14] Delamarre L, Gollion C, Grouteau G, Rousset D, Jimena G, Roustan J (2020). COVID-19-associated acute necrotising encephalopathy successfully treated with steroids and polyvalent immunoglobulin with unusual IgG targeting the cerebral fibre network. J Neurol Neurosurg Psychiatry.

[15] Deshmukh DR, Maassab HF, Mason M (1982). Interactions of aspirin and other potential etiologic factors in an animal model of Reye syndrome. Proc Natl Acad Sci USA.

[16] Dixon L, Varley J, Gontsarova A, Mallon D, Tona F, Muir D (2020). COVID-19-related acute necrotizing encephalopathy with brain stem involvement in a patient with aplastic anemia. Neurol Neuroimmunol Neuroinflamm.

[17] Dos Santos BR, de Melo Jorge DM, Castro IA, Moretto EL, Scalon de Oliveira L, Ubiali EMA (2020). Detection of Influenza A(H3N2) Virus RNA in Donated Blood. Emerg Infect Dis.

[18] Flewett TH, Hoult JG (1958). Influenzal encephalopathy and postinfluenzal encephalitis. Lancet.

[19] Frankova V, Jirasek A, Tumova B (1977). Type A influenza: postmortem virus isolations from different organs in human lethal cases. Arch Virol.

[20] Frobert E, Sarret C, Billaud G, Gillet Y, Escuret V, Floret D (2011). Pediatric neurological complications associated with the A(H1N1)pdm09 influenza infection. J Clin Virol.

[21] Furuta Y, Gowen BB, Takahashi K, Shiraki K, Smee DF, Barnard DL (2013). Favipiravir (T-705), a novel viral RNA polymerase inhibitor. Antiviral Res.

[22] Goenka A, Michael BD, Ledger E, Hart IJ, Absoud M, Chow G (2014). Neurological manifestations of influenza infection in children and adults: results of a National British Surveillance Study. Clin Infect Dis.

[23] Gooskens J, Kuiken T, Claas EC, Harinck HI, Thijssen JC, Baelde HJ (2007). Severe influenza resembling hemorrhagic shock and encephalopathy syndrome. J Clin Virol.

[24] Han T, Lai Y, Jiang Y, Liu X, Li D (2021). Influenza A virus infects pulmonary microvascular endothelial cells leading to microvascular leakage and release of pro-inflammatory cytokines. PeerJ.

[25] Hase Y, Horsburgh K, Ihara M, Kalaria RN (2018). White matter degeneration in vascular and other ageing-related dementias. J Neurochem.

[26] Hasegawa S, Matsushige T, Inoue H, Shirabe K, Fukano R, Ichiyama T (2011). Serum and cerebrospinal fluid cytokine profile of patients with 2009 pandemic H1N1 influenza virus-associated encephalopathy. Cytokine.

[27] Hatachi T, Michihata N, Inata Y, Takeuchi M, Matsui H, Fushimi K (2021). Prognostic factors among children with acute encephalitis/encephalopathy associated with viral and other pathogens. Clin Infect Dis.

[28] Hidaka F, Matsuo S, Muta T, Takeshige K, Mizukami T, Nunoi H (2006). A missense mutation of the Toll-like receptor 3 gene in a patient with influenza-associated encephalopathy. Clin Immunol.

[29] Hiyoshi M, Indalao IL, Yano M, Yamane K, Takahashi E, Kido H (2015). Influenza A virus infection of vascular endothelial cells induces GSK-3beta-mediated beta-catenin degradation in adherens junctions, with a resultant increase in membrane permeability. Arch Virol.

[30] Hochberg FH, Nelson K, Janzen W (1975). Influenza type B-related encephalopathy. The 1971 outbreak of Reye syndrome in Chicago. JAMA.

[31] Hoshino A, Saitoh M, Oka A, Okumura A, Kubota M, Saito Y (2012). Epidemiology of acute encephalopathy in Japan, with emphasis on the association of viruses and syndromes. Brain Dev.

[32] Hosoya M, Nunoi H, Aoyama M, Kawasaki Y, Suzuki H (2005). Cytochrome c and tumor necrosis factor-alpha values in serum and cerebrospinal fluid of patients with influenza-associated encephalopathy. Pediatr Infect Dis J.

[33] Howard A, Uyeki TM, Fergie J (2018). Influenza-Associated acute necrotizing encephalopathy in siblings. J Pediatric Infect Dis Soc.

[34] Ichiyama T, Morishima T, Isumi H, Matsufuji H, Matsubara T, Furukawa S (2004). Analysis of cytokine levels and NF-kappaB activation in peripheral blood mononuclear cells in influenza virus-associated encephalopathy. Cytokine.

[35] Ichiyama T, Morishima T, Kajimoto M, Matsushige T, Matsubara T, Furukawa S (2007). Matrix metalloproteinase-9 and tissue inhibitors of metalloproteinases 1 in influenza-associated encephalopathy. Pediatr Infect Dis J.

[36] Imakita N, Kitabatake M, Ouji-Sageshima N, Hara A, Morita-Takemura S, Kasahara K (2019). Abrogated Caveolin-1 expression via histone modification enzyme Setdb2 regulates brain edema in a mouse model of influenza-associated encephalopathy. Sci Rep.

[37] Ishigami A, Kubo S, Ikematsu K, Kitamura O, Tokunaga I, Gotohda T (2004). An adult autopsy case of acute encephalopathy associated with influenza A virus. Leg Med (Tokyo).

[38] Ishige T, Igarashi Y, Hatori R, Tatsuki M, Sasahara Y, Takizawa T (2018). IL-10RA mutation as a risk factor of severe influenza-associated encephalopathy: a case report. Pediatrics.

[39] Ito Y, Torii Y, Ohta R, Imai M, Hara S, Kawano Y (2011). Increased levels of cytokines and high-mobility group box 1 are associated with the development of severe pneumonia, but not acute encephalopathy, in 2009 H1N1 influenza-infected children. Cytokine.

[40] Jeganathan N, Fox M, Schneider J, Gurka D, Bleck T (2013). Acute hemorrhagic leukoencephalopathy associated with influenza A (H1N1) virus. Neurocrit Care.

[41] Jeong JH, Choi WS, Antigua KJC, Choi YK, Govorkova EA, Webby RJ (2020). In vitro profiling of laninamivir-resistant substitutions in N3 to N9 avian influenza virus neuraminidase subtypes and their association with in vivo susceptibility. J Virol.

[42] Kasai M, Shibata A, Hoshino A, Maegaki Y, Yamanouchi H, Takanashi JI (2020). Epidemiological changes of acute encephalopathy in Japan based on national surveillance for 2014–2017. Brain Dev.

[43] Kawashima H, Morishima T, Togashi T, Yokota S, Yamanaka G, Ioi H (2004). Extraordinary changes in excitatory amino acid levels in cerebrospinal fluid of influenza-associated encephalopathy of children. Neurochem Res.

[44] Kawashima H, Nishimata S, Suzuki S (2008). Pathology of pediatric infectious immunological diseases-influenza-associated encephalopathy-. Byori to Rinsho.

[45] Kimura-Ohba S, Yang Y, Thompson J, Kimura T, Salayandia VM, Cosse M (2016). Transient increase of fractional anisotropy in reversible vasogenic edema. J Cereb Blood Flow Metab.

[46] Kimura-Ohba S, Asaka MN, Utsumi D, Takabatake Y, Takahashi A, Yasutomi Y (2023). d-Alanine as a biomarker and a therapeutic option for severe influenza virus infection and COVID-19. Biochim Biophys Acta Mol Basis Dis.

[47] Kobayashi Y, Kanazawa H, Hoshino A, Takamatsu R, Watanabe R, Hoshi K (2019). Acute necrotizing encephalopathy and a carnitine palmitoyltransferase 2 variant in an adult. J Clin Neurosci.

[48] Krol E, Rychlowska M, Szewczyk B (2014). Antivirals–current trends in fighting influenza. Acta Biochim Pol.

[49] Kuiken T, Taubenberger JK (2008) Pathology of human influenza revisited. Vaccine 26(Suppl 4):D59–D66 10.1016/j.vaccine.2008.07.025 10.1016/j.vaccine.2008.07.025 PMC2605683 19230162

[50] Kuki I, Shiomi M, Okazaki S, Kawawaki H, Tomiwa K, Amo K (2015). Characteristic neuroradiologic features in hemorrhagic shock and encephalopathy syndrome. J Child Neurol.

[51] Kuki I, Inoue T, Nukui M, Okazaki S, Kawawaki H, Ishikawa J (2022). Longitudinal electroencephalogram findings predict acute neurological and epilepsy outcomes in patients with hemorrhagic shock and encephalopathy syndrome. Epilepsy Res.

[52] Kyan Y, Ueda Y, Yoshida M, Sasahara K, Shinya K (2014). Transcriptome profiling of brain edemas caused by influenza infection and lipopolysaccharide treatment. J Med Virol.

[53] Lee LYY, Zhou J, Frise R, Goldhill DH, Koszalka P, Mifsud EJ (2020). Baloxavir treatment of ferrets infected with influenza A(H1N1)pdm09 virus reduces onward transmission. PLoS Pathog.

[54] Lyon JB, Remigio C, Milligan T, Deline C (2010). Acute necrotizing encephalopathy in a child with H1N1 influenza infection. Pediatr Radiol.

[55] Majde JA, Kapas L, Bohnet SG, De A, Krueger JM (2010). Attenuation of the influenza virus sickness behavior in mice deficient in Toll-like receptor 3. Brain Behav Immun.

[56] Manley GT, Fujimura M, Ma T, Noshita N, Filiz F, Bollen AW (2000). Aquaporin-4 deletion in mice reduces brain edema after acute water intoxication and ischemic stroke. Nat Med.

[57] Maricich SM, Neul JL, Lotze TE, Cazacu AC, Uyeki TM, Demmler GJ (2004). Neurologic complications associated with influenza A in children during the 2003–2004 influenza season in Houston. Texas Pediatrics.

[58] Mizuguchi M, Abe J, Mikkaichi K, Noma S, Yoshida K, Yamanaka T (1995). Acute necrotising encephalopathy of childhood: a new syndrome presenting with multifocal, symmetric brain lesions. J Neurol Neurosurg Psychiatry.

[59] Mori I, Komatsu T, Takeuchi K, Nakakuki K, Sudo M, Kimura Y (1995). Viremia induced by influenza virus. Microb Pathog.

[60] Mori I, Goshima F, Imai Y, Kohsaka S, Sugiyama T, Yoshida T (2002). Olfactory receptor neurons prevent dissemination of neurovirulent influenza A virus into the brain by undergoing virus-induced apoptosis. J Gen Virol.

[61] Morichi S, Yamanaka G, Ishida Y, Oana S, Kashiwagi Y, Kawashima H (2014). Brain-derived neurotrophic factor and interleukin-6 levels in the serum and cerebrospinal fluid of children with viral infection-induced encephalopathy. Neurochem Res.

[62] Morichi S, Morishita N, Takeshita M, Ishida Y, Oana S, Yamanaka G (2017). Vascular endothelial growth factor (VEGF) and platelet-derived growth factor (PDGF) levels in the cerebrospinal fluid of children with influenza-associated encephalopathy. J Infect Chemother.

[63] Morishima T, Togashi T, Yokota S, Okuno Y, Miyazaki C, Tashiro M (2002). Encephalitis and encephalopathy associated with an influenza epidemic in Japan. Clin Infect Dis.

[64] Morita A, Ishihara M, Kamei S, Okuno H, Tanaka-Taya K, Oishi K (2019). Nationwide survey of influenza-associated acute encephalopathy in Japanese adults. J Neurol Sci.

[65] Muradrasoli S, Mohamed N, Belak S, Czifra G, Herrmann B, Berencsi G (2010). Broadly targeted triplex real-time PCR detection of influenza A, B and C viruses based on the nucleoprotein gene and a novel “MegaBeacon” probe strategy. J Virol Methods.

[66] Nagaoka Y, Nosaka N, Yamada M, Yashiro M, Washio Y, Baba K (2017). Local and systemic immune responses to influenza a virus infection in pneumonia and encephalitis mouse models. Dis Markers.

[67] Nakai Y, Itoh M, Mizuguchi M, Ozawa H, Okazaki E, Kobayashi Y (2003). Apoptosis and microglial activation in influenza encephalopathy. Acta Neuropathol.

[68] Namba T, Tsuge M, Yashiro M, Saito Y, Liu K, Nishibori M (2021). Anti-high mobility group box 1 monoclonal antibody suppressed hyper-permeability and cytokine production in human pulmonary endothelial cells infected with influenza A virus. Inflamm Res.

[69] Nara A, Nagai H, Yamaguchi R, Yoshida K, Iwase H, Mizuguchi M (2015). An unusual autopsy case of cytokine storm-derived influenza-associated encephalopathy without typical histopathological findings: autopsy case report. Am J Forensic Med Pathol.

[70] Nogusa S, Thapa RJ, Dillon CP, Liedmann S, Oguin TH, Ingram JP (2016). RIPK3 activates parallel pathways of MLKL-driven necroptosis and FADD-mediated apoptosis to protect against influenza A virus. Cell Host Microbe.

[71] Ohashi E, Hayakawa I, Murofushi Y, Kawai M, Suzuki-Muromoto S, Abe Y (2021). Recurrent acute necrotizing encephalopathy in a boy with RANBP2 mutation and thermolabile CPT2 variant: the first case of ANE1 in Japan. Brain Dev.

[72] Okumura A, Nakagawa S, Kawashima H, Morichi S, Muguruma T, Saito O (2013). Severe form of encephalopathy associated with 2009 pandemic influenza A (H1N1) in Japan. J Clin Virol.

[73] Okuno H, Yahata Y, Tanaka-Taya K, Arai S, Satoh H, Morino S (2018). Characteristics and outcomes of influenza-associated encephalopathy cases among children and adults in Japan, 2010–2015. Clin Infect Dis.

[74] Omoto S, Speranzini V, Hashimoto T, Noshi T, Yamaguchi H, Kawai M (2018). Characterization of influenza virus variants induced by treatment with the endonuclease inhibitor baloxavir marboxil. Sci Rep.

[75] Oughton M, Dascal A, Laporta D, Charest H, Afilalo M, Miller M (2011). Evidence of viremia in 2 cases of severe pandemic influenza A H1N1/09. Diagn Microbiol Infect Dis.

[76] Papadopoulos MC, Manley GT, Krishna S, Verkman AS (2004). Aquaporin-4 facilitates reabsorption of excess fluid in vasogenic brain edema. FASEB J.

[77] Petullo D, Masonic K, Lincoln C, Wibberley L, Teliska M, Yao DL (1999). Model development and behavioral assessment of focal cerebral ischemia in rats. Life Sci.

[78] Poyiadji N, Shahin G, Noujaim D, Stone M, Patel S, Griffith B (2020). COVID-19-associated acute hemorrhagic necrotizing encephalopathy: imaging features. Radiology.

[79] Saitoh M, Shinohara M, Hoshino H, Kubota M, Amemiya K, Takanashi JL (2012). Mutations of the SCN1A gene in acute encephalopathy. Epilepsia.

[80] Sanchez-Lanier M, Davis LE, Blisard KS, Woodfin BM, Wallace JM, Caskey LS (1991). Influenza A virus in the mouse: hepatic and cerebral lesions in a Reye’s syndrome-like illness. Int J Exp Pathol.

[81] Shinohara M, Saitoh M, Nishizawa D, Ikeda K, Hirose S, Takanashi J (2013). ADORA2A polymorphism predisposes children to encephalopathy with febrile status epilepticus. Neurology.

[82] Shinya K, Makino A, Hatta M, Watanabe S, Kim JH, Hatta Y (2011). Subclinical brain injury caused by H5N1 influenza virus infection. J Virol.

[83] Solis-Garcia G, Chacon-Pascual A, Gonzalez Martinez F, Miranda Herrero MC, Hernandez-Sampelayo T, Catalan Alonso P (2020). Neurologic complications in children hospitalized with influenza infections: prevalence, risk factors and impact on disease severity. Pediatr Infect Dis J.

[84] Sumikoshi M, Hashimoto K, Kawasaki Y, Sakuma H, Suzutani T, Suzuki H (2008). Human influenza virus infection and apoptosis induction in human vascular endothelial cells. J Med Virol.

[85] Swierczynska M, Mirowska-Guzel DM, Pindelska E (2022). Antiviral drugs in influenza. Int J Environ Res Public Health.

[86] Tachibana M, Mohri I, Hirata I, Kuwada A, Kimura-Ohba S, Kagitani-Shimono K (2019). Clasmatodendrosis is associated with dendritic spines and does not represent autophagic astrocyte death in influenza-associated encephalopathy. Brain Dev.

[87] Tada H, Takanashi J, Barkovich AJ, Oba H, Maeda M, Tsukahara H (2004). Clinically mild encephalitis/encephalopathy with a reversible splenial lesion. Neurology.

[88] Takahashi M, Yamada T, Nakashita Y, Saikusa H, Deguchi M, Kida H (2000). Influenza virus-induced encephalopathy: clinicopathologic study of an autopsied case. Pediatr Int.

[89] Takanashi J, Oba H, Barkovich AJ, Tada H, Tanabe Y, Yamanouchi H et al (2006) Diffusion MRI abnormalities after prolonged febrile seizures with encephalopathy. Neurology 66(9):1304–1309; discussion 1291. 10.1212/01.wnl.0000210487.36667.a5. 10.1212/01.wnl.0000210487.36667.a5 16682659

[90] Tanaka T, Sunden Y, Sakoda Y, Kida H, Ochiai K, Umemura T (2010). Lipopolysaccharide treatment and inoculation of influenza A virus results in influenza virus-associated encephalopathy-like changes in neonatal mice. J Neurovirol.

[91] Tetsuhara K, Akamine S, Matsubara Y, Fujii S, Kashimada W, Marutani K et al (2022) Severe encephalopathy associated with SARS-CoV-2 Omicron BA.1 variant infection in a neonate. Brain Dev 44(10):743–747. 10.1016/j.braindev.2022.06.010 10.1016/j.braindev.2022.06.010 PMC9273474 35835638

[92] Togashi T, Matsuzono Y, Narita M, Morishima T (2004). Influenza-associated acute encephalopathy in Japanese children in 1994–2002. Virus Res.

[93] Tsuge M, Yasui K, Ichiyawa T, Saito Y, Nagaoka Y, Yashiro M (2010). Increase of tumor necrosis factor-alpha in the blood induces early activation of matrix metalloproteinase-9 in the brain. Microbiol Immunol.

[94] Virhammar J, Kumlien E, Fallmar D, Frithiof R, Jackmann S, Skold MK (2020). Acute necrotizing encephalopathy with SARS-CoV-2 RNA confirmed in cerebrospinal fluid. Neurology.

[95] Wabuke-Bunoti MA, Bennink JR, Plotkin SA (1986). Influenza virus-induced encephalopathy in mice: interferon production and natural killer cell activity during acute infection. J Virol.

[96] Wang PY, Yang MT, Liang JS (2022). Acute necrotizing encephalopathy caused by SARS-CoV-2 in a child. Pediatr Neonatol.

[97] Wang G, Zhang J, Li W, Xin G, Su Y, Gao Y (2008). Apoptosis and proinflammatory cytokine responses of primary mouse microglia and astrocytes induced by human H1N1 and avian H5N1 influenza viruses. Cell Mol Immunol.

[98] Watanabe C, Kawashima H, Takekuma K, Hoshika A, Watanabe Y (2008). Increased nitric oxide production and GFAP expression in the brains of influenza A/NWS virus infected mice. Neurochem Res.

[99] Weisheng H, Hui Z, Shuang W, Qing G, Tianying S, Chenguang Y (2022). A sudden death due to acute necrotizing encephalopathy associated with influenza A virus infection: an autopsy case report. Leg Med (Tokyo).

[100] Wells CE (1971) Neurological complications of so-called “influenza”. A winter study in South-east Wales. Br Med J 1(5745):369–373. 10.1136/bmj.1.5745.369 10.1136/bmj.1.5745.369 PMC1794999 4322460

[101] Yao D, Kuwajima M, Chen Y, Shiota M, Okumura Y, Yamada H (2007). Impaired long-chain fatty acid metabolism in mitochondria causes brain vascular invasion by a non-neurotropic epidemic influenza A virus in the newborn/suckling period: implications for influenza-associated encephalopathy. Mol Cell Biochem.

[102] Zhao C, Gan Y, Sun J (2011). Radiographic study of severe Influenza-A (H1N1) disease in children. Eur J Radiol.

