# Supplementary material for: Viral entry and translation in brain endothelia provoke influenza-associated encephalopathy
Source: Acta Neuropathol. 2024 Apr 30;147(1):77. doi: 10.1007/s00401-024-02723-z (PMC11061015; doi:10.1007/s00401-024-02723-z)
Supplement: Supplementary file 1 — Supplementary file1 (PDF 384 KB) [file 401_2024_2723_MOESM1_ESM.pdf]

Supplementary Information

Viral entry in brain endothelia and viral translation provoke influenza-associated encephalopathy

Shihoko Kimura-Ohba, Mieko Kitamura, Yusuke Tsukamoto, Shigetoyo Kogaki, Shinsuke Sakai, Hiroaki Fushimi, Keiko Matsuoka, Makoto Takeuchi, Kyoko Itoh, Keiji Ueda, Tomonori Kimura

Supplementary Methods

Antibodies

Anti-mouse CD31 (CD31; 1:50, Dianova), influenza A virus nucleoprotein antibody (fluA-NP; 1:1600, Gene Tex), anti-CD31 antibody [C31.3+JC/70A] (CD31; 1:100, Abcam), anti-Iba-1 (1:3000, Wako), MLKL polyclonal antibody (MLKL; 1:100, Proteintech), phospho- mixed lineage kinase domain-like protein antibody (p-MLKL; 1:50, Invitrogen), anti-caspase-8 antibody (1:50, Abcam), anti-glial fibrillary acidic protein antibody (GFAP; 1:1000, Abcam), anti-GFAP [Clone G-A-5] (GFAP;1:500, Sigma-Aldrich), aquaporin-4 antibody (AQP4; 1:5000, Thermo Fisher Scientific), and goat anti mouse IgG H&L (Alexa Fluor®594) (1:500, Abcam).

Viral quantitative analysis with animal brain tissue and infected cells

The supernatant of either cells or brain tissue in 10-folds dilution series was applied to MDCK cells seeded in 6-well plates at approximately 95% confluency, 6-well plates were then incubated for 1 h at 37℃ with rocking every 15 min. The inoculum was removed, and the cells were washed twice with PBS. MDCK cells were then covered with overlay media (serum-free MEM containing 2 mmol/l L-glutamine with 0.75% Agarose ME (Iwai Chemicals Company Ltd.)) and incubated at 37℃ for 72 h. MDCK cells were fixed and stained with 1% crystal violet to count the number of plaques. Infectious titers of the supernatant were defined according to the number of plaque-forming units (pfu)/ml, the dilution ratio of the supernatant, and the tissue weights (fluid volume (ml) for the whole blood).

Supplementary Table.1

	Case1	Case2	Case3
Age	13y	5y	1y
Died at	2004	1998	1998
Diagnosis of IAE	HSES	Reye’s syndrome	Reye’s syndrome
Co-morbidities	Tuberous sclerosis	Not reported	CHARGE association
Viral protein detectection in brain	Yes	Yes	Yes
Viral protein colocalized at EC	Yes	Yes	Yes

Supplementary Figures

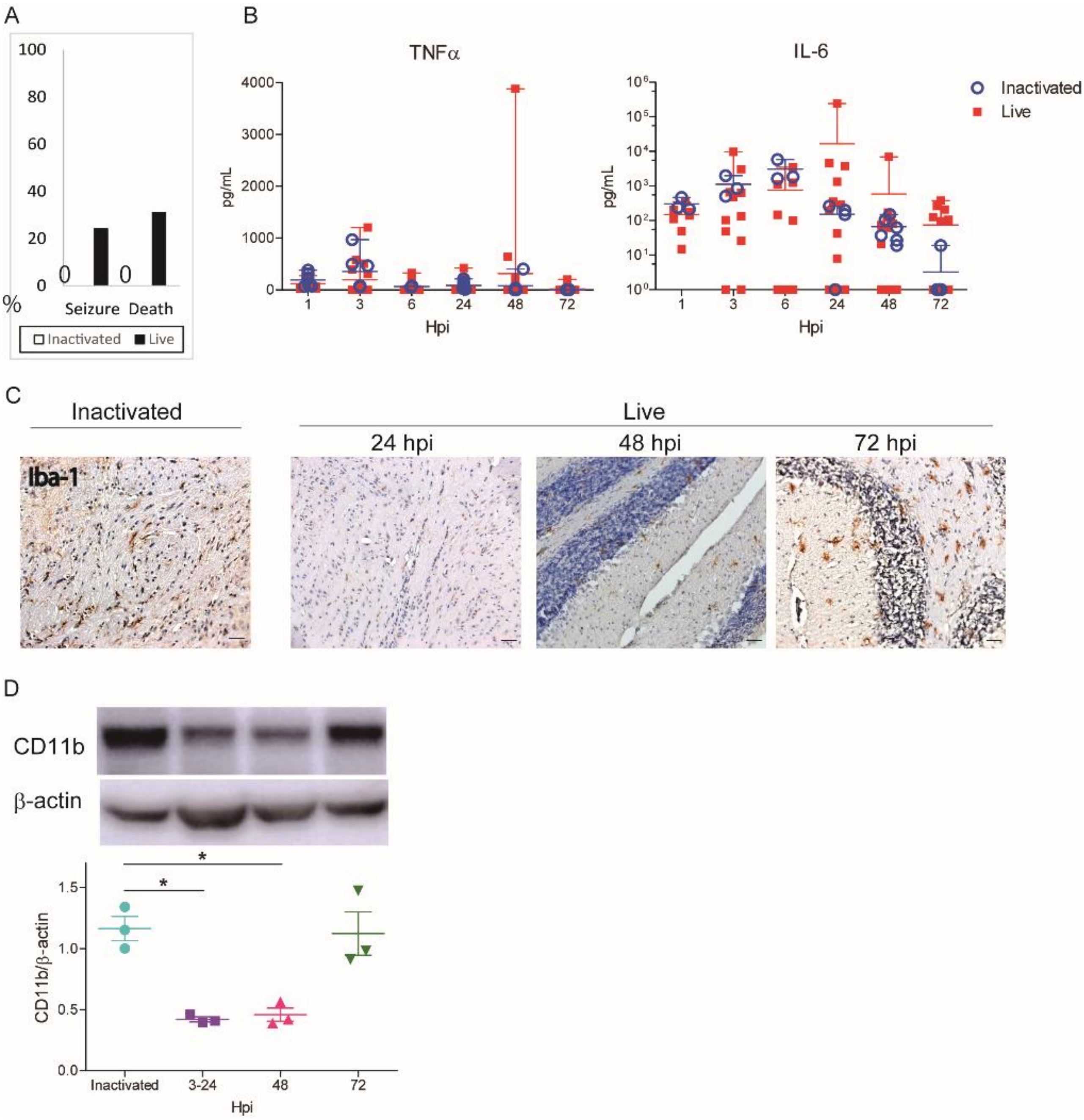

Supplementary Figure 1. **The IVE mouse model mimicked the characteristics of the patients with IAE.**

(A) Seizure and death rates of IVE mice. (B) Cytokine levels (left, TNFα; right, IL-6) of IVE mice serum harvested at the indicated hpi. (C) Representative images of the brains of IVE mice harvested at the indicated hpi stained with Iba-1 (a marker of microglia). (D) Quantification of CD11b (a marker of microglia) by western blot from the brains of IVE mice at the indicated hpi. *n* = 3

Scale bars indicate (C) 50 μm. Data are presented as means ± SE, *p < 0.05.

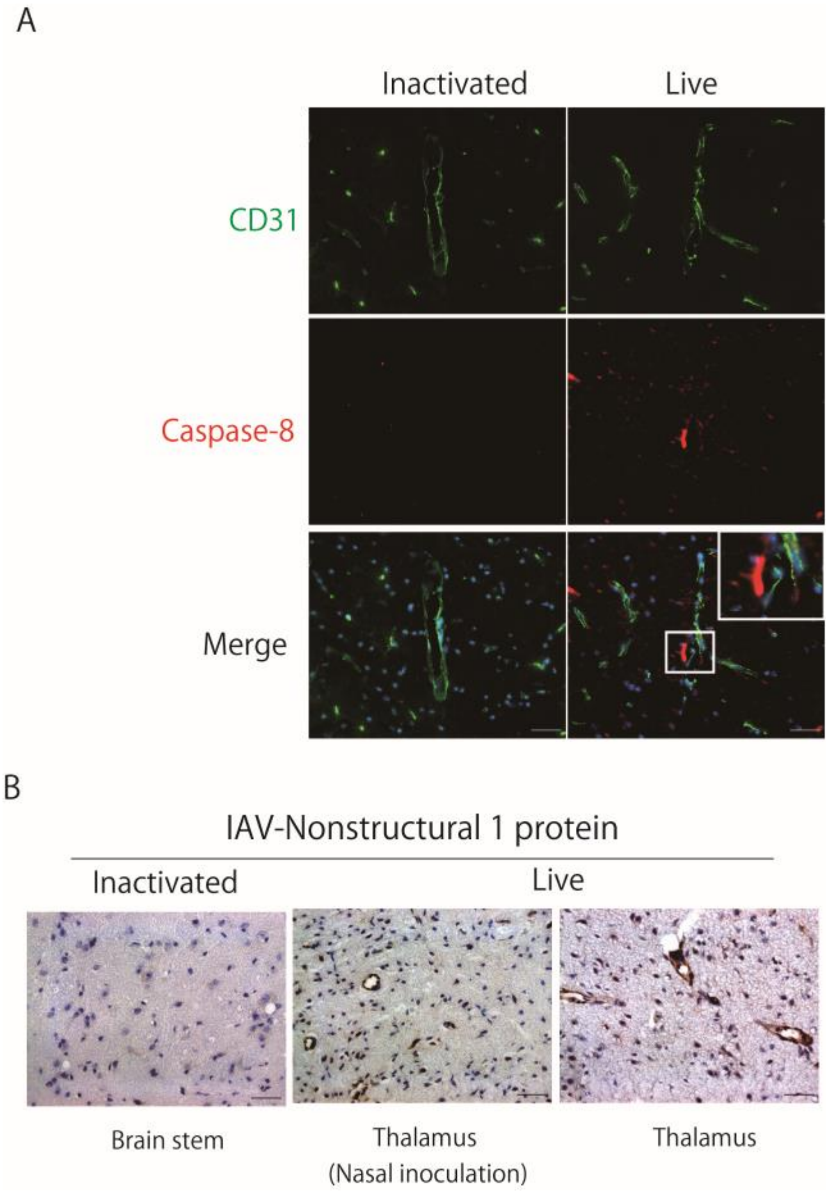

Supplementary Figure 2. Histological analysis of mouse brains infected with IAV.

(A) Images of the brains of IVE mice at 72 hpi stained with anti-caspase-8 antibody. Red, Caspase-8 (a marker of apoptosis); green, CD31; blue, DAPI; *n* = 3. (B) Representative images of brains from mice with inactivated (left panel) and live (middle and right panels) IAV intranasal inoculation, harvested at 120 hpi and stained with IAV-nonstructural 1 (NS-1) protein.

Scale bars indicate 50 µm,

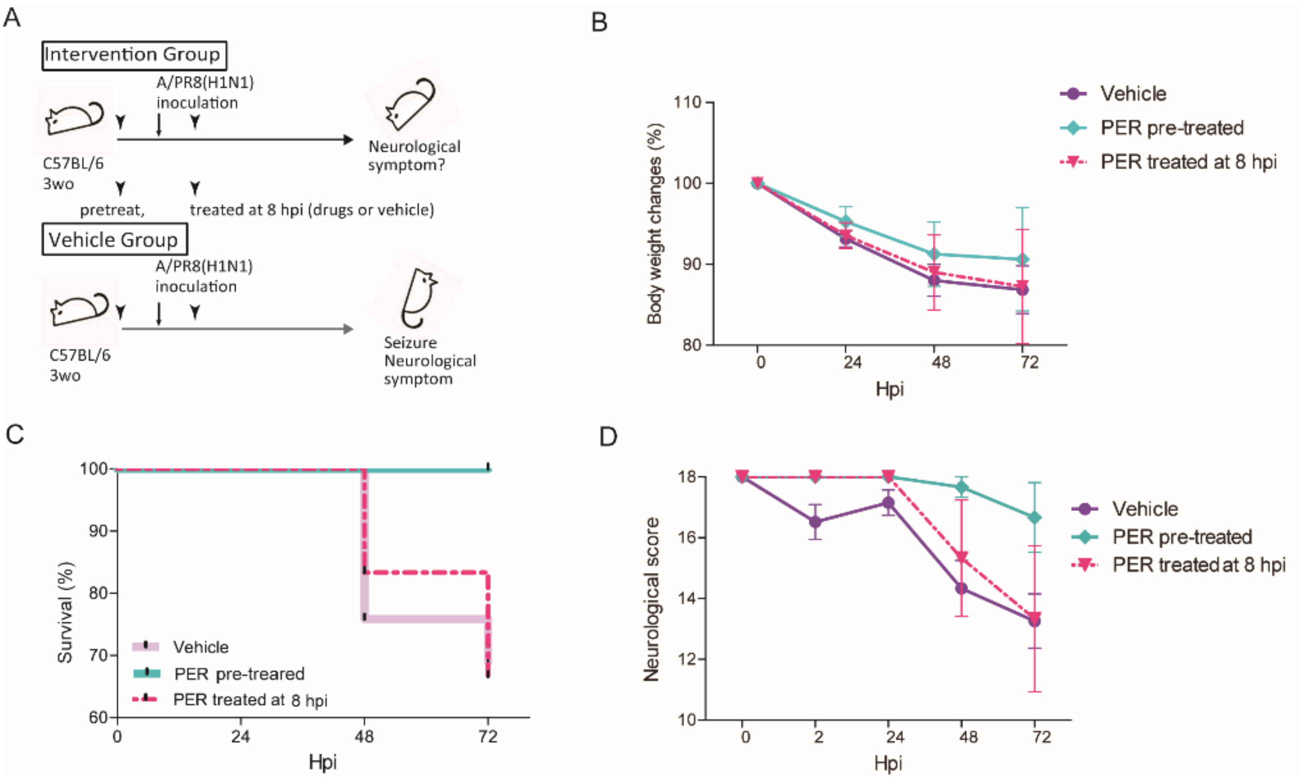

Supplementary Figure 3. Improved lethality of the IVE mice by a neuraminidase inhibitor.

(A) Schematic diagram of the experimental schedule. IVE mice were treated with PER, either before (pre-treatment) or 8 h after inoculation (post-treatment). (B) Change in body weight, (C) Kaplan-Meier analysis of survival rate, and (D) neurological scores of IVE mice. *n* = 6–11.
